# Predominant Non-additive Effects of Multiple Stressors on Autotroph C:N:P Ratios Propagate in Freshwater and Marine Food Webs

**DOI:** 10.3389/fmicb.2018.00069

**Published:** 2018-01-30

**Authors:** Manuel Villar-Argaiz, Juan M. Medina-Sánchez, Bopaiah A. Biddanda, Presentación Carrillo

**Affiliations:** ^1^Departamento de Ecología, Facultad de Ciencias, Universidad de Granada, Granada, Spain; ^2^Annis Water Resources Institute, Grand Valley State University, Muskegon, MI, United States; ^3^Instituto del Agua, Universidad de Granada, Granada, Spain

**Keywords:** interactive effects, C:N:P ratios, stoichiometry, autotroph, microbial loop, zooplankton

## Abstract

A continuing challenge for scientists is to understand how multiple interactive stressor factors affect biological interactions, and subsequently, ecosystems–in ways not easily predicted by single factor studies. In this review, we have compiled and analyzed available research on how multiple stressor pairs composed of temperature (T), light (L), ultraviolet radiation (UVR), nutrients (Nut), carbon dioxide (CO_2_), dissolved organic carbon (DOC), and salinity (S) impact the stoichiometry of autotrophs which in turn shapes the nature of their ecological interactions within lower trophic levels in streams, lakes and oceans. Our analysis from 66 studies with 320 observations of 11 stressor pairs, demonstrated that non-additive responses predominate across aquatic ecosystems and their net interactive effect depends on the stressor pair at play. Across systems, there was a prevalence of antagonism in freshwater (60–67% vs. 47% in marine systems) compared to marine systems where synergism was more common (49% vs. 33–40% in freshwaters). While the lack of data impeded comparisons among all of the paired stressors, we found pronounced system differences for the L × Nut interactions. For this interaction, our data for C:P and N:P is consistent with the initial hypothesis that the interaction was primarily synergistic in the oceans, but not for C:N. Our study found a wide range of variability in the net effects of the interactions in freshwater systems, with some observations supporting antagonism, and others synergism. Our results suggest that the nature of the stressor pairs interactions on C:N:P ratios regulates the “continuum” commensalistic-competitive-predatory relationship between algae and bacteria and the food chain efficiency at the algae-herbivore interface. Overall, the scarce number of studies with even more fewer replications in each study that are available for freshwater systems have prevented a more detailed, insightful analysis. Our findings highlighting the preponderance of antagonistic and synergistic effects of stressor interactions in aquatic ecosystems—effects that play key roles in the functioning of feedback loops in the biosphere—also stress the need for further studies evaluating the interactive effects of multiple stressors in a rapidly changing world facing a confluence of tipping points.

## Introduction

“*An ecosystem is greater than the sum of its parts*.”

–Eugene P. Odum (1964)

A paramount theme in the scientific and political arena is to better understand and predict the impact of human activities on the functioning of ecosystems, such as modification of biogeochemical cycles, climate change or species harvest and biodiversity loss (Carpenter et al., 2008; Cheung et al., 2009). We have greatly advanced our knowledge about the molecular and cellular basis of these impacts, but are still blind to the effects on the higher levels of integration of populations and ecosystems. Much of the higher complexity that arises as we scale up to higher trophic levels is a consequence of the interaction of species within their biotic and abiotic environment (Krebs, 2009; Boyd and Hutchins, 2012). While ecologists have traditionally focused on a particular level of integration (population, community, or ecosystem ecology), modern ecology is no longer viewed as isolated parts anymore, instead seeks to theoretically connect all levels of integration. One theoretical approach that has greatly advanced our knowledge on this systematic integration is Ecological Stoichiometry (ES). In short, ES is the scientific study of the balance of multiple chemical elements in ecological interactions (Sterner and Elser, 2002). Although, it can be lightly classified as a highly reductionistic approach, implications of stoichiometry span from atoms to all higher levels of integration: growth and reproduction rates, nutrient recycle, interspecific interactions or food quality and energy transfer in food webs are all subject to the limitations imposed by the availability and stoichiometry of elements. A core pillar of stoichiometry is that chemical composition is variable and reflects that of the available substrate in plants but is relatively tight in their consumer heterotrophs (Sterner and Elser, 2002). Herbivores with high somatic demands for nutrients face a world where plants are for the most part poor food quality resources (Hessen, 1992). As a result, nutrients and energy that flow through consumer-resource interactions obey to the fundamental constraints of a mass balance reaction and thermodynamics, with a myriad of consequences for organism's growth, population dynamics and ecological processes (Sterner and Elser, 2002).

Global change is happening now and has already affected numerous species and ecosystem processes (Pace et al., 2015). Relevant global factors include increased atmospheric CO_2_ with consequences for global warming, alterations in biochemical cycles (e.g., N and P), local and regional eutrophication, habitat use and land use alterations or increased UV radiation, among others. A fundamental strength of ES in the study of global change is that the multiplicity of human activities and natural perturbations have an impact at the base of food webs because the elemental composition of algae and plants in general, reflect the resource availability in their environment, and can be traced as they indirectly influence secondary producers and predators at higher levels (Sterner and Elser, 2002). In fact, numerous observations suggest that the indirect-food chain mediated effects of a stressor can be far more significant than direct effects on organisms at any given trophic level (e.g., Durif et al., 2015). There is an increasing awareness among scientists that realistic scenarios of global changes need holistic approaches that include multiple interactive factors (Folt et al., 1999). For example, current climate change due to rising CO_2_ concentrations are likely having an effect on photosynthetic rates in plants. At the same time, the associated rise in temperature can stimulate growth of many species, but enhance stratification of the water column, which can itself exacerbate nutrient exhaustion in surface waters leading to community changes and greater sensitivity to photosynthesis and UVR. Organisms are exposed to multiple stressors simultaneously, whose interactions can enhance or decrease the effect of a given stressor (Folt et al., 1999; Crain et al., 2008; Boyd et al., 2015). However, while numerous studies have documented how environmental conditions affect the elemental composition of primary producers, there is far less information on the combined effects of multiple global stressors, and even fewer studies have examined the role of stoichiometry on how multiple interactive effects impact ecological interactions. If examining the role of interactive effects on stoichiometry is not a trivial task, establishing the carry-on consequences of these effects on ecological interactions is even more difficult as it demands thorough work explicitly gauging cause-effect relationships. Studies on this incipient research area would be immensely valuable in our path to an improved predictive framework for interactive effects in nature.

The initial convention distinguished between interactions that increased stress or “synergistic” from those that decreased stress or “antagonistic” (Folt et al., 1999; Gunderson et al., 2016). While the study of interactions has been extremely active of late [see for example recent reviews by Jackson et al. (2015) and Piggott et al. (2015)], still a consensus is lacking in the literature regarding an operational definition of interactions. Our intention in this study is not to examine the appropriateness of a given classification method for the effects of multiple stressors. Instead, we adhered to a basic definition of synergism and antagonism to illustrate the nature of the net effect of multiple stressors in ecosystems with the purpose of improving our understanding of the mechanisms behind multiple stress effects and the habitat-specific prevalence of the various types of interactions in nature. On the basis of a literature survey, Crain et al. (2008) and Jackson et al. (2015) found that while the effect of paired stressors were consistently antagonistic or additive in freshwater systems, there was a greater prevalence of synergistic interactions in marine systems.

In this review, we hierarchically examine progress in these areas of ecological interactions and stoichiometry by answering several fundamentally related questions: What are the interactive effects of multiple stressors on the elemental composition of primary producers in aquatic ecosystems? Are there habitat differences in the prevalence of synergistic, antagonistic or additive responses? To address these questions, we will first compile field and laboratory investigations that examine the effect of paired stressors on the elemental stoichiometry of autotrophs in aquatic ecosystems and classify them as synergistic or antagonistic according to a modification of Allgeier et al.'s interaction effect index (IEI) (Allgeier et al., 2011), which compares the cumulative mean size effect of two paired stressors with the sum of their individual effects (see methods for further explanation). With the resulting database, we will test whether patterns in the prevalence of a given interaction differ across systems and, more specifically, the general hypothesis that interactive effects across paired stressors are primarily synergistic in marine systems, but antagonistic or additive in freshwater ecosystems.

Finally, we discuss recent progress about the relevance of the effects of paired environmental drivers in fundamental food web relationships (microbial loop, algae-herbivore), identify gaps in the research of multiple stressors, and suggest new avenues for stressor interactions research.

## Data extraction and statistical analyses

Experimental literature on the impacts of multiple stressors on C:N:P ratios was searched using Web of Science (review to January 2017). The following criteria were applied to select articles:

1. Published studies on the research topic were identified using the following search keywords: “stoichiometry,” “interactive effect^*^,” “C:N^*^,” and “C:P^*^.” For each extracted study, we examined the cited references in search of additional suitable data, and used ResearchGate and Google Scholar as supplementary research tools in addition to Web of Science.
2. The dataset covered laboratory and *in situ* experiments in which the effects of paired stressors on the C:N:P composition of the autotrophs in aquatic food webs (plankton, epilithon or periphyton) were reported. Macrophytes were excluded from this analysis. As in Crain et al. (2008), a stressor was defined in a broad sense as any environmental global driver that can potentially exceed natural levels of variation.
3. The study belonged to any of habitat categories of streams, lakes (reservoirs or lakes) and marine systems, regardless of whether these were field or controlled laboratory studies using species from their natural habitats.

**Figure 1:**
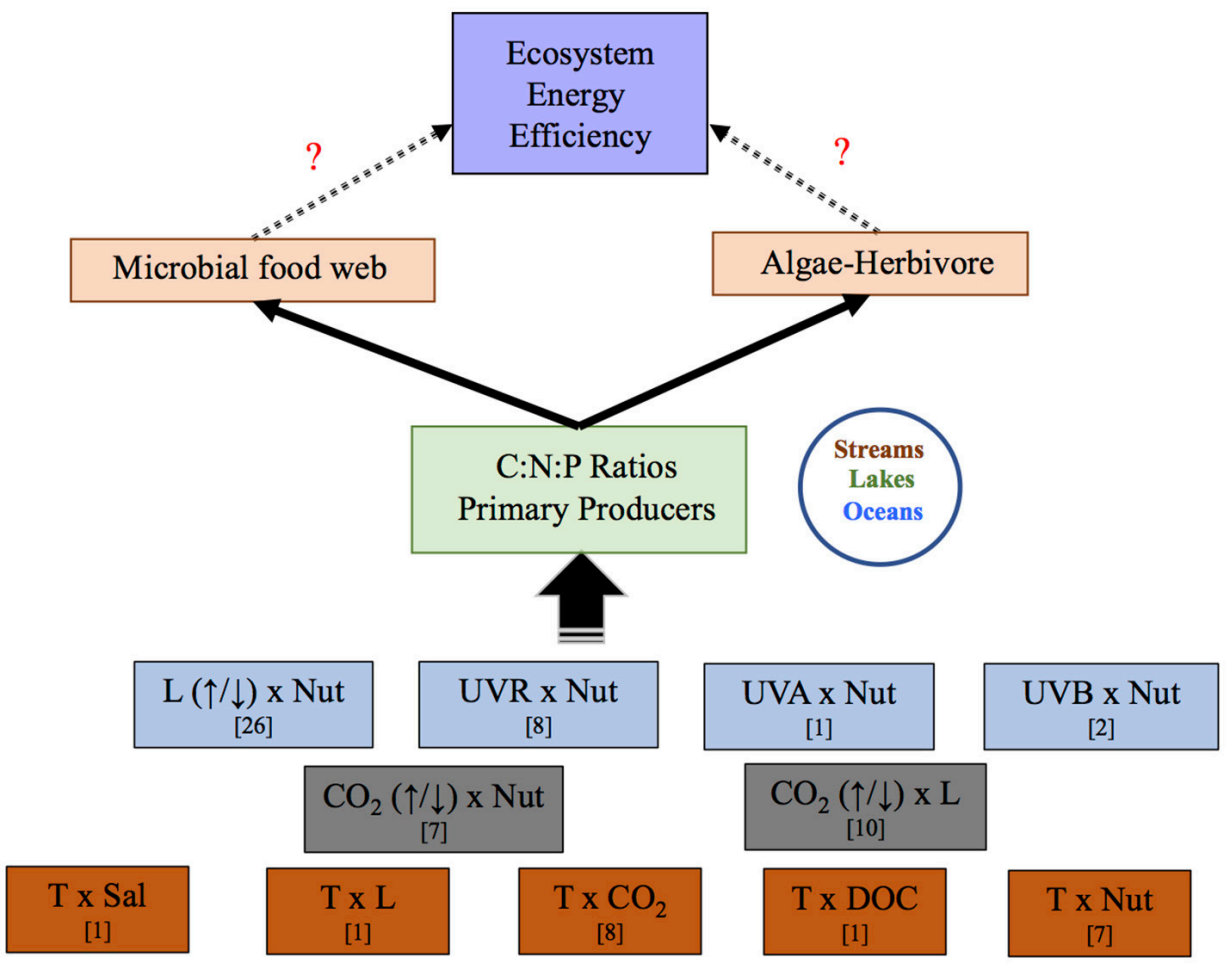
Scheme illustrating the potential effects of stressor pairs in food web interactions via changes in C:N:P stochiometry. Eleven stressor-pairs were analyzed in this study. L, light; Nut, nutrients; UVR, ultraviolet radiation; T, temperature; Sal, salinity; CO_2_, carbon dioxide; DOC, dissolved organic carbon. Question marks indicate pathways lacking information. In brackets is number of studies found for each stressor pair.

Sixty-six studies met the study selection criteria outlined above (full list in Table S1). Our database compiled a total of 320 observations, since most studies reported more than one observation. We investigated a total of 11 stressor pairs (Figure 1). Whenever possible, we obtained C:N:P values from the result section of the study. A PlotDigitizer software (Free Software Foundation, 2001) was used to acquire numbers from the figures when data were not readily available in the results. Care was taken to differentiate between observations testing the interactive effects of an increase or decrease in the main stressor. For example, studies covering L × Nut effects on C:N:P ratios were classified in two broad categories depending on whether experiments tested the increase (↑L × Nut) by supplementing light conditions or decrease (↓L × Nut) by shading or reducing light supplementation relative to that received by the organisms in their habitat (field research) or optimal level (laboratory assays). Also decreased or increased stressor scenarios could help differentiate between future and past scenarios in the various ratios, as well as future opposed predictions for a given stressor. For example, most studies agree upon lower-than-today CO_2_ levels (~150 ppm) for past glacial events and higher-than-today CO_2_ levels for future CO_2_ scenarios (500–1,000 ppm), although there is the potential for future scenarios of deprived CO_2_ in local environments of markedly increased photosynthesis.

To illustrate the overall nature of the interactive effects of multiple stressor on C:N:P ratios, we modified the Interaction Effect Index (IEI) proposed by Allgeier et al. (2011) according to the following equation:

IEI = ln [Abs(effect AB/(effect A + effect B))]

where A and B are the two stressors, “Abs” indicates absolute value, “effect AB” is the combined effect of AB calculated as (treatment_A+B_−control), “effect A” is the main effect of A calculated as (treatment_A_−control), and “effect B” is the main effect of B calculated as (treatment_B_−control).

**Figure 2:**
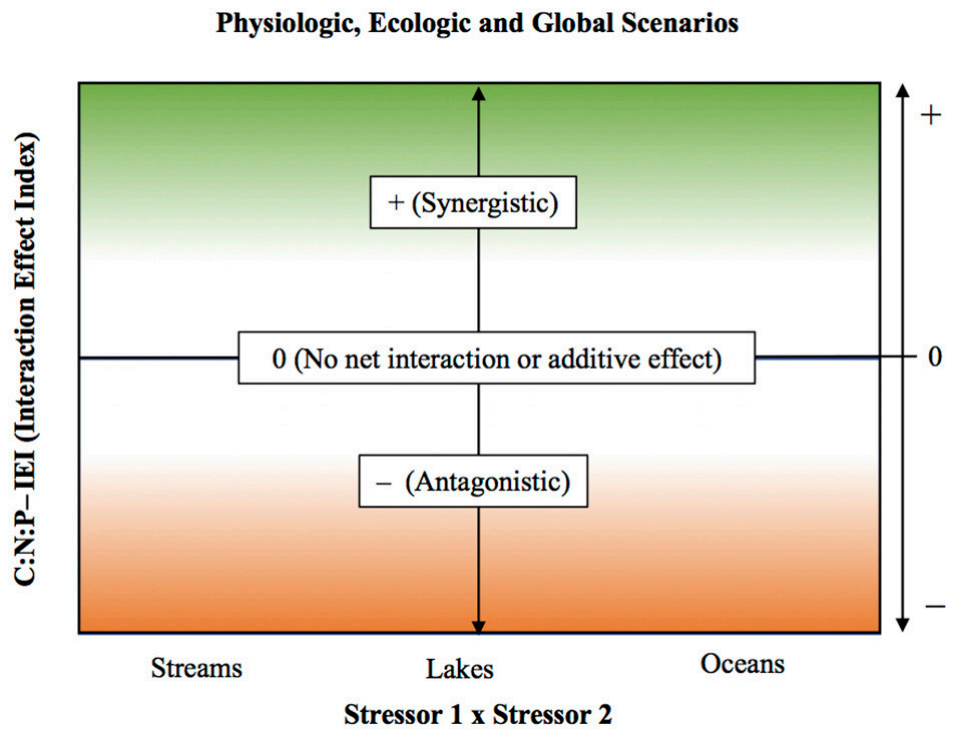
Scheme illustrating the interpretation of the Interactive Effect Index (IEI) for each stressor pair. IEI was calculated as Ln [ABS (Combined/Additive effects)] (see statistical methods for details). The IEI in the y-axis reflects the nature of the interactive effect for C:N:P ratios, with values > 0 indicating a synergistic interaction, values < 0 indicating an antagonistic interaction, and values = 0 indicating the absence of a net interactive effect or the occurrence of an additive effect. Stressor pairs are shown in x-axis.

Taking the effect ratio as the natural logarithm of the absolute quotient between combined (effect AB) and additive effects (effect A + effect B), has important advantages compared to other forms of evaluating interactive effects: (i) it allows calculation of IEI for all integer numbers (negative, zero, and positive numbers), (ii) includes standardization of the responses, what reduces variability in the interactive effects and grants the use of parametric analysis for comparative purposes, and (iii) centers IEI values around zero. We used IEI in this study to identify the three broad categories of interactions types (as defined in Folt et al., 1999) on C:N:P ratios (see Figure 2). IEI values above one were defined as cases in which the interaction was synergistic, whereas IEI values below one indicated antagonistic interaction, whereas values non distinguishable from zero indicated the occurrence of an additive effect or the absence of a net interactive effect.

**Figure 3:**
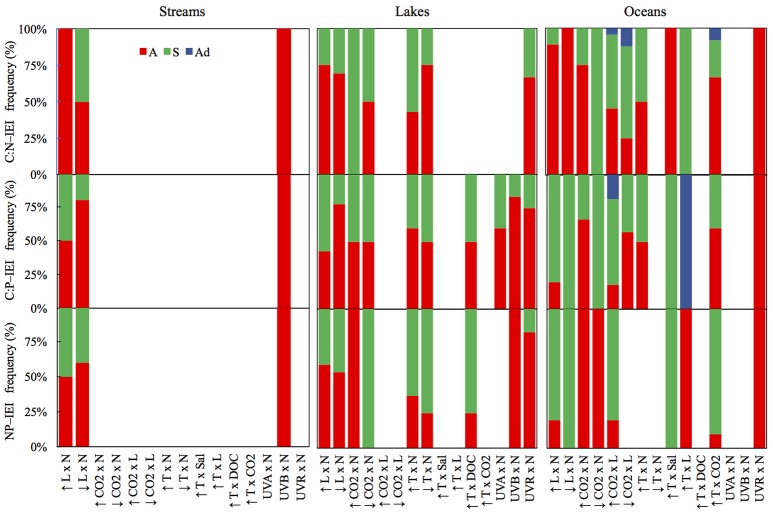
Mean frequencies (%) of C:N:P-IEI interaction types for streams, lakes and oceans for the different stressor pairs tested in this study.

**Figure 4:**
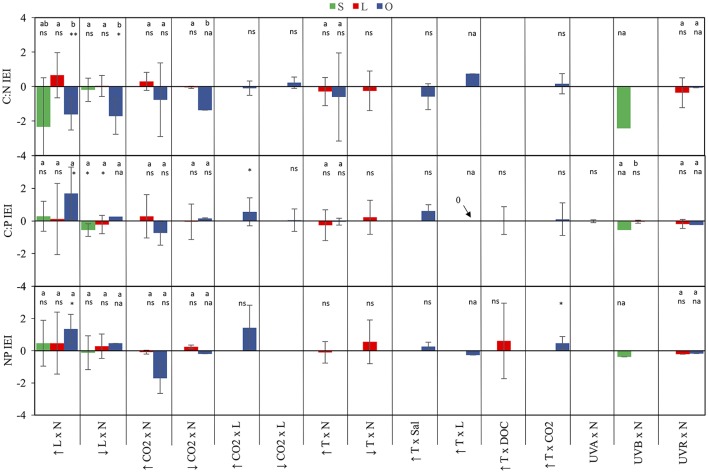
IEI for C:N:P ratios in streams, lakes and oceans. Values represent IEI mean ± 95% confidence intervals (see statistical methods for further detail). Values > 0 indicate a synergistic interaction, values < 0 indicate an antagonistic interaction, and values = 0 indicate the absence of a net interactive effect or the occurrence of an additive effect. Significant differences among systems are denoted by different case letters according to Tuckey's *post*-*hoc* comparisons. Below letters and/or symbols are results for one-sample *t*-test testing IEI differences from the test value = 0 (ns, not significant; na, not applicable with less than two observations; ^*^p < 0.05; ^**^p < 0.01).

The frequency of interaction types was calculated for the various subsets corresponding with the C:N, C:P, and N:P ratios in the studied species. This graphic representation of these data in Figure 3 contributed to identify differences in the prevailing interaction type among systems and C:N:P ratios. Finally, one-sample *t*-tests were used to evaluate whether mean C:N:P-IEI differed from zero for each stressor pair and system (Figure 4). If the null hypothesis is rejected, it demonstrates an interactive effect (antagonisms or synergisms). On the other hand, non-rejection of the null hypothesis was interpreted as the absence of significant interaction, which does not necessarily mean the presence of an additive effect because observations with opposing interactive effects could compensate each other such that their mean might not significantly differ from zero.

Significant differences in C:N:P-IEI between habitats and drivers were tested using one-way ANOVA, followed by *post*-*hoc* comparison of the means using Tukey's HSD (Sokal and Rohlf, 1995). Normality was test by Kolmogorov-Smirnov test and homocedasticity by Cochran's and Levene's tests. Although care was taken to differentiate data that originally tested interactions in our illustrations (see different bar colors in Figures S1–S6), we chose to include all observations in our intersystem comparison in order to increase the statistical confidence of our findings. IBM SPSS Statistics (version 24) was used for all data analyses and a statistical significance level of *p* < 0.05 was applied to all tests.

## Interactive effects of global change stressors on autotroph C:N:P ratios

### Light × nutrients

The key role of light and nutrients in the structure and functioning of ecosystems was first identified in a benchmark contribution by Lindeman (1942). Since then, numerous experimental and observational studies have shown that the production of algae and biomass of pelagic herbivores increases with nutrient enrichment (Murdoch et al., 1998). Aquatic autotrophs might also “compete” for the light availability. Therefore, the nutrient-phytoplankton coupled dynamics as well as changes in the mixing depth due to global warming are expected to have important consequences for the light availability in the upper layers of the water column (Carrillo et al., 2015). We now know that light and nutrient availability not only affect production of algae but also their C:nutrient ratio. This idea was elegantly formulated by Sterner et al. (1997) as the Light:nutrient hypothesis which predicts that low light conditions decrease not only production in primary producers but also their C:nutrient ratio, and potentially offsets the negative effects of decreased food availability for their pelagic consumers. Simultaneous algal controlled assays provided with experimental evidence for the chief role of these two factors on the elemental composition of phytoplankton (Urabe and Sterner, 1996). In addition, global change research has reported strong changes in the biogeochemical cycles of N, P, and C (Falkowski et al., 2000) and predict changes in the solar radiation underwater mainly due to increased temperature or land alterations and droughts that might regionally increase dust emissions to the atmosphere (IPCC, 2013). Therefore, anthropogenic impacts on ecosystems can drive changes in light and nutrient availability with consequences for the elemental composition of plants at the base of food webs.

Light and nutrients was the only stressor pair for which we found sufficient evidence to compare interactive effect among streams, lakes and oceans (Figure S1). While most observations consisted of antagonistic and synergistic effects, there were clear differences in IEI among habitats and C:N:P ratios (Figure 3). In an scenario of increased light and nutrients, IEI varied among habitats in C:N ratio, but not in C:P and N:P ratios (Figure 3, Table 1). More specifically, *Post-hoc* comparisons indicated that the effect on C:N-IEI was different between lakes and oceans, but neither of these habitats differed from streams (Figure 4). In agreement with our general hypothesis of synergism prevalence in oceanic systems, IEI was synergistic for C:P and N:P ratios in the oceans, but not for C:N. As for streams and lakes, IEI did not statistically differ from zero, which was a consequence of the low number of evidences (*n* = 2 in rivers) or a situation where stressor effects were opposing directions (i.e., synergism and antagonism).

**Table 1:** Results of ANOVA testing whether interactive effects for C:N:P ratios [ln (combined effect/additive effects)] differ across systems (streams, lakes and oceans).

**Interactive effect**	**Factor 1**	**C: nutrient**	**d.f**.	**Sum of squares**	***F***	***p*-value**
L × Nut	↑ Light	C:N	**2, 13**	**20.77**	**4.80**	**0.028**
		C:P	2, 11	7.60	1.05	0.383
		N:P	2, 9	2.24	0.41	0.677
	↓ Light	C:N	**2, 15**	**11.26**	**5.31**	**0.012**
		C:P	2, 20	1.23	0.43	0.658
		N:P	2, 14	0.61	1.20	0.323
CO_2_ × Nut	↑ CO_2_	C:N	1, 2	0.06	0.016	0.912
		C:P	1, 2	2.12	2.35	0.265
		N:P	1, 2	2.69	10.69	0.082
	↓ CO_2_	C:N	**1, 1**	**1.21**	**297.78**	**0.037**
		C:P	1, 2	0.04	0.071	0.815
		N:P	1, 1	0.13	27.00	0.121
T × Nut	↑ T	C:N	1, 7	0.17	0.11	0.748
		C:P	1, 10	0.07	0.08	0.778
		N:P	–	–	–	–
UVB × Nut	UVB	C:N	–	–	–	–
		C:P	**1, 4**	**0.17**	**16.30**	**0.016**
		N:P	–	–	–	–
UVR × Nut	UVR	C:N	1, 2	0.16	0.28	0.648
		C:P	1, 8	0.02	0.42	0.535
		N:P	1, 3	0.001	0.012	0.920

*Only stressor pairs with observations for at least two systems (streams, lakes, oceans) were included in this analysis. In bold are significant differences*.

As for the scenario of increased light, the effect of interactive effects of decreased light × nutrients on C:N deviated from our initial prediction and was also antagonistic in oceans. As for C:P and N:P ratios, we had no statistically discernible effects with a single study that yielded a positive synergism (Figure 3). As expected, interactive effects in both stream and lakes were either additive for C:N and N:P or antagonistic for C:P (Figure 4).

### CO_2_ × nutrients

Climate change due to increased CO_2_ is the most notable feature of global change. The extra CO_2_ can directly fuel photosynthesis worldwide and indirectly impact the C:N:P stoichiometry of plankton by decreasing the nutrient content of autotrophs (increased C:P and C:N ratios) at the base of trophic webs (Riebesell et al., 2007; Verschoor et al., 2013). Elevated CO_2_ concentrations only seem to change phytoplankton stoichiometry under specific conditions, for instance, at low nutrient availability (Gervais and Riebesell, 2001; Leonardos and Geider, 2005; Li et al., 2012) whereas rising CO_2_ levels will increase phytoplankton biomass at high nutrient loads (Verspagen et al., 2014). The enrichment of aquatic systems with anthropogenic CO_2_ is already having consequences on acidification and nutrient biogeochemistry (Gattuso et al., 2015). Since biogeochemical cycles are intrinsically linked, the change in carbon will have large consequences on the nitrogen cycle through microbial mediated processes such as increases in N fixation or denitrification and decreases in nitrification (Hutchins et al., 2009). At the same time, humans are altering planet's biogeochemical cycles at unprecedented rates (Falkowski et al., 2000). Consequently, the net impact of human on biochemical cycles may change ratios of nutrients at big scales that can unbalance the stoichiometry of ecological interactions and alter natural and managed ecosystems across the globe (Peñuelas et al., 2013). Anthropogenic alterations of N and P biochemical cycles are nearly 10 and 40-fold higher those of C cycle (Falkowski et al., 2000). Because the availability of N limits primary production in much of the ocean (Moore et al., 2013) and continental waters (Elser et al., 2007), human activities might be responsible for much of the nitrogen fertilization via N_2_O emissions and industrial, agriculture and wastewater discharges (Duce et al., 2008). Similarly, there has been an increase in P inputs in the biosphere due to mining of P compounds for fertilizer (Falkowski et al., 2000). While there is uncertainty on whether the higher mobilization of essential nutrient can boost up primary production and help mitigate CO_2_ accumulation in Earth's atmosphere (Falkowski et al., 2000), there is still no conclusive evidence of whether the combined effects of increased CO_2_ and nutrients would lead to increase or decrease in C:N:P ratios (Hutchins et al., 2009).

Only few studies in lakes and oceans covered the effects of this stressor pair. In the scenario of increased CO_2_ there was a general prevalence of synergistic effects in lakes but antagonistic in oceans (Figures 3, 4). The only two studies to statistically test the increased CO_2_ × Nut effect were carried out in the ocean and were consistent with the general appreciation for the prevalence of antagonistic effects in the ocean, but only for the C:P and N:P ratio (Figure S2). Not surprisingly, IEI did not differ from zero in most comparisons due to the low number of observations.

While most studies to date examine the effects of future CO_2_ levels, low CO_2_ concentration represents a potential scenario for past glacial ages where this gas diminished on a global scale (Sigman and Boyle, 2000). Also decreased CO_2_ availability is a likely future scenario in sites where nutrient eutrophication could boost autotroph growth depriving the concentration of this photosynthetic resource. Under the scenario of decreased CO_2_, there was an antagonistic interaction on C:N (Table 1), but indistinguishable from zero in the rest of the cases, indicating that the effect of decreased CO_2_ on C:nutrient ratio was not necessarily affected by nutrient availability (Figures 3, 4).

### CO_2_ × light

Phytoplankton have an essential role in sequestering CO_2_ at global scales, and therefore play a key role in the partitioning of CO_2_ between the atmosphere and the hydrosphere (Leonardos and Geider, 2005). However, the capacity of the ocean as a carbon sink is determined not only by CO_2_ levels, but also by other environmental variables that affect photosynthesis CO_2_ fixation such as nutrient availability or light. Light conditions can drastically vary in the future due to global changes in the upper mixed layer depth (Carrillo et al., 2015), and a crucial question is whether this might exacerbate or mitigate the effects of increasing CO_2_ in the physiology of phytoplankton.

For this stressor pair, only ocean observations were available in the literature. The prevailing interactions for both the scenarios of increased and decreased CO_2_ were synergistic, although some additive observations were observed (Figure S3, Figure 3). Overall, the synergistic interactions were statistically significantly different from zero only for C:P ratio in the scenario of increased CO_2_, but indistinguishable from additive effect for the rest of comparisons (Figure 4).

### Temperature × nutrients

Recent unprecedented increase in atmospheric CO_2_ is responsible for warming of lake and oceanic surface waters around the globe (Schlüter et al., 2014). Mean temperatures and heat waves that are expected to increase can have profound effects on autotrophs and heterotrophs, and thus ecosystem functioning (IPCC, 2013). Temperature is an all-embracing environmental factor that affects the growth, reproduction and survival of organisms, and the interactions among species (Kingsolver, 2009). Rising temperatures *per ser* can have an effect on organisms chemical composition (Woods et al., 2003), and favor the dense blooms of toxic cyanobacteria (Johnk et al., 2008). Additionally, higher temperatures can alter ambient conditions by reducing the duration of ice (IPCC, 2013), decreasing lake water levels (Hanrahan et al., 2010), changing patterns in phenology (Menzel et al., 2006) and reducing nutrient fluxes from the hypolimnion due to the greater stability of the stratification process (Huisman et al., 2006). The study of the joint effect of temperature and nutrients on autotrophs is of particular importance. First, these are two factors where pronounced global changes have already been detected (IPCC, 2013). Second, evidence exists that warming can reduce the effects of eutrophication on periphyton by altering C:N ratios (Shurin et al., 2012). However, while nutrient availability may decrease in C:N ratio, this effect could be counterbalanced by depletion of nutrients due to strengthening stratification as a result of global warming (van de Waal et al., 2010).

For this stressor pair, the small sample size only allowed for the comparison of differential responses on C:N and C:P between lakes and oceans in the scenario of increased temperature, in which no differences were detected (Table 1). In addition, IEI values for most ratios were equally distributed between antagonistic and synergistic cases (Figure S4, Figure 3), resulted in values of mean IEI that did not significantly differ from zero (Figure 4).

### Temperature × other stressors

Future shifts in the chemical composition of algae are also anticipated given the continuous changes in environmental drivers that interact with temperature (Passow and Laws, 2015). For example, some studies have documented temperature pair interactions with salinity, light or CO_2_ on the stoichiometry of marine phytoplankton yielding contrasting responses. Thus, for the temperature salinity stressor pair, the effects were antagonistic for C:N, but synergistic for C:P and N:P (Figure S5, Figure 3). As for the only study covering temperature and light effects, the interactions switched from synergistic for C:N to additive for C:P and antagonistic for N:P (Figure S5, Figure 3). We identified eight studies that focus on the temperature-CO_2_ stressor pair in the ocean, which yielded a similar distribution of synergistic and antagonistic responses for C:N and C:P ratios, and a dominant, and significant synergistic response for N:P ratio (Figures 3, 4, Table 1). Only one study in lakes covered the interactive effects of increased temperature and DOC availability yielding inconclusive findings for the nature of the interactions on the algal stoichiometry due to the low number of observations and their opposing interactive signs (Figure 3).

### UVR × nutrients

Compared to photosynthetically active radiation (PAR), exposure to UVR increases C:nutrient ratios in algae (Xenopoulos et al., 2002; Carrillo et al., 2008a; Korbee et al., 2012). As a result, the net outcome of the two opposing effects of UVR and PAR on algal composition depends on the optical properties of the water column (Hessen et al., 2008), as well as factors like nutrients that promote phytoplankton growth influencing the light climate in the water column.

Although empirical evidence for the nature of the combined effects of UVR radiation and nutrients on elemental ratio of autotrophs remains very limited, the compiled dataset allowed us to distinguish among the interactive effects of distinct spectral regions of UVR and nutrients. Thus, the combined effects of UVA and nutrients on algal C:P ratios in lakes, frequencies of interactions varied between synergistic and antagonistic (Figure S6, Figure 3). The only stream observation in the dataset for the effects of UVB and nutrients yielded antagonistic effects on all C:N:P ratios (Figure 3), which differed from the additive effect found for C:P in lakes (Table 1). Finally, the type of interactions for UVR paired with nutrients did not significantly differ between lakes and oceans and were predominantly antagonistic (Figures 3, 4, Table 1).

## Propagation of interactive effects on food webs

As we have seen, few studies have evaluated the impact of multiple stressors on the elemental composition of planktonic organisms (Table S1), but barely a handful of them examined the carry-on consequences of multiple interactive effects on the role of stoichiometry on ecological interactions. Unraveling the “ecological surprises” (*sensu* Jackson et al., 2015) that arise from the interactive impacts of multiple environmental stressors on C:N:P is a thrilling challenge that can help understand ecological responses to environmental change. In this section we will examine the few available studies to assess how multiple stressors impact ecological interactions within the microbial and grazing food chains via stoichiometry.

### Microbial food web

The coevolution of algae and bacteria has shaped life on Earth in many aspects and their interaction has, not only defined the structure of their habitats (Ashen and Goff, 2000), but is responsible for the productivity of the biosphere (Ramanan et al., 2016). Numerous lines of evidence indicate that the long coevolution of algae and bacteria has given rise to diverse types of associations including mutualism, commensalism, competition or parasitism (Ramanan et al., 2016, and references therein). A significant step in the study of the relationship between algae and bacteria is the insight that their complex association points to a relation continuum, where the relationship between organisms transits from a commensal (one organism benefits with no detriment to the partner's fitness) to mutualistic (two organisms that benefit one another) to predatory/parasitic (one organisms benefits at the expense of the other) relationship. But what is the role of C:N:P ratios in shaping this continuum?

A first level of approach stems from one-factor experimental studies adding nutrients at definite N:P ratios in highly UVR-exposed systems, which changed algal and bacterial N:P ratios and modulated their interaction with consequences for the development of the microbial loop (Carrillo et al., 2008b). Variations in algal and bacterial N:P ratios revealed a shift from their commensalistic-mutualistic relationship to a competitive one for the available P when bacterial P-deficiency increased (N:P > 20–24). Hence, the bacterial N:P ratio proved to be a key factor in understanding the algae—bacteria relationship. The development of ciliates occurred only when bacteria remained P-rich (N:P < 20) and algae was close to Redfield proportions indicating that bacterial N:P ratio also served as an essential predictor for the ciliate and microbial loop development (Villar-Argaiz et al., 2002; Carrillo et al., 2008b).

**Figure 5:**
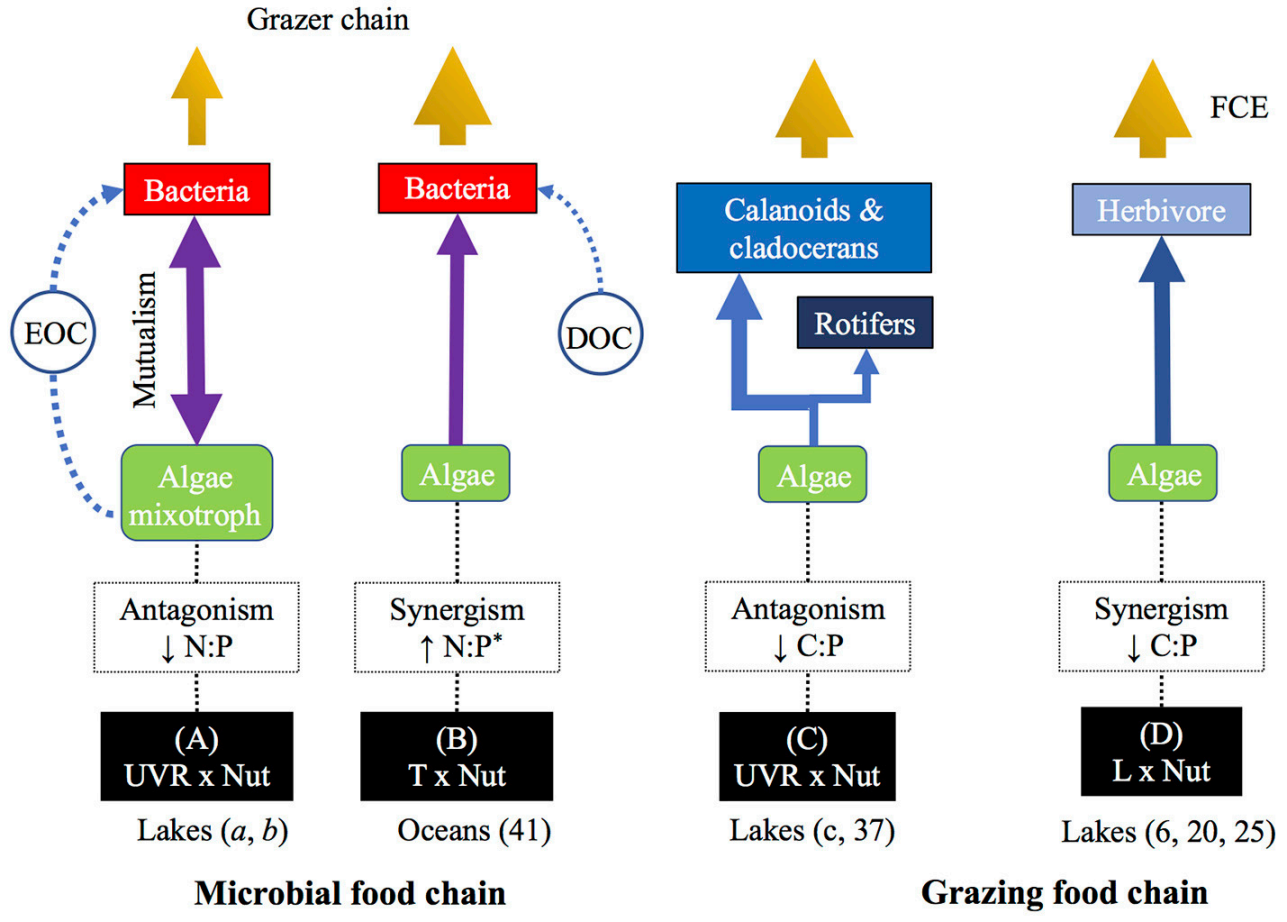
Conceptual diagram illustrating the nature of the interactive effect between **(A)** UVR × Nut, **(B)** T × Nut, **(C)** UVR × Nut, and **(D)** L × Nut on autotroph C:N:P ratios and its propagation in microbial and grazing food chains. The figure summarizes the main food web effects analyzed in this study. In parenthesis are studies cited in the manuscript (*a*, Medina-Sánchez et al., 2004; *b*, Cabrerizo et al., 2017; *c*, Villar-Argaiz et al., 2012) or supplementary references (41, 6, 20, and 25). Arrow thinkness is proportional to magnitude of the effect. EOC, excretion of organic carbon, DOC, dissolved organic carbon, FCE, food chain efficiency. ^(^*^)^Type of interactive effect calculated for N:P ratio from Figure 2 in reference 41.

A second level of approach stems from two-factor experimental studies manipulating resource N:P (by adding nutrients at definite N:P ratios) and another global-change stressor. Thus, Medina-Sánchez et al. (2006) set up three resource N:P treatments (N:P > 180, N:P = 16, and N:P = 5) in presence vs. absence of UVR in two contrasting unbalanced scenarios in the algal and bacterial N:P ratio in a high mountain lake (i.e., middle of ice-free period: P-poor algae and P-sufficient bacteria; late ice-free period: P-rich algae and P-poor bacteria). Under such scenarios, these authors found antagonistic UVR × P effects (i.e., the effect of P enrichment attenuated the effect of UVR) that decreased autotroph N:P ratio, increased the excretion of organic carbon by algae, and in turn reinforced the commensalistic-predatory relationship between algae and bacteria (Figure 5A). This dual control exerted by mixotrophic algae implies a shift in the paradigm of the functioning of microbial food web in lakes (Medina-Sánchez et al., 2004; Cabrerizo et al., 2017), recently extended to marine ecosystems (Ptacnik et al., 2016). In a different experiment, Wohlers-Zöllner et al. (2011) set up two resource N:P treatments (N:P = 9 as “N-deficient” vs. N:P = 30 as “N-replete”) and a temperature gradient simulating weak to strong warming predicted for winter in the Baltic Sea region at 2100. The pelagic algal-bacterial assemblage showed a synergistic interactive effect on autotroph N:P ratio which enhanced utilization of organic matter and bacterial growth and, ultimately, reinforced the development of the microbial food web (Figure 5B). These studies illustrate the key role of resource stoichiometry in determining how paired stressors modulate the interaction between algae and bacteria.

A third level of approach stems from experimental two-factor studies, manipulating UVR (presence vs. absence) and nutrient availability in mid to long term scales (days to months) and reporting pronounced changes in the autotrophs stoichiometry (N:P). For example, Delgado-Molina et al. (2009) showed that the antagonistic interaction between UVR and nutrients generated a gradual decrease in the algal N:P ratio that ultimately leads to unimodal responses in the heterotrophic microbial food web (bacteria, ciliates, and viruses), where the effect of UVR is maximum at intermediate P concentrations (Medina-Sánchez et al., 2013).

### Grazing food web

The plant-herbivore interface is the level at which nutrient imbalances are among the highest in nature (Sterner and Elser, 2002), constraining the energy transfer and production of higher trophic levels in food webs (Power, 1992). Several potential factors affect the food quality for herbivores, and research has extensively addressed the carryover consequences of single stressors, primarily nutrient availability, on herbivore performance mediated through changes in plant chemical composition. To date, most research has covered freshwater species (DeMott et al., 1998; Villar-Argaiz and Sterner, 2002), but also included stream (Stelzer and Lamberti, 2002) and marine species (Schoo et al., 2010). We examine the few studies to systematically evaluate the interactive effect of paired stressors at the plant-herbivore interface.

#### Food quality × temperature

Several studies have examined the joint role of food quality, as cultured algae to a nutrient replete or limited state, and temperature on zooplankton performance (McFeeters and Frost, 2011; Persson et al., 2011; Malzahn et al., 2016). These works generally agreed upon the conclusion that low food quality constraints on herbivore growth were strongest at low temperatures and decreased at high temperatures. In other words, the highest herbivore growth at low diet C:P ratios (high P availability) and elevated temperature demonstrated that P food quality and temperature synergistically increased herbivore performance. Studies like these, provide a very valuable perspective on how temperature can indirectly affect species worldwide by enhancing their likelihood to face P limitation.

#### Temperature × DOC

One test of the importance of multiple stressors on herbivore communities comes from the study by Weidman et al. (2014) in alpine lakes, where zooplankton were expected to respond strongly to increased water temperature. The effects of temperature × DOC on particulate C:P ratios were antagonistic on these lakes (see reference 46 in Figure S5). As for the carryover effects on zooplankton, while warming alone stimulated the growth of the cladoceran and suppressed that of the calanoid copepod, the combined effect of warming and DOC reversed these results. Therefore, the addition of DOC suppressed the detrimental effect of temperature on copepod and total zooplankton biomass. These findings imply that the antagonistic temperature × DOC effects resulted in food changes that contributed to overcome the negative effect of temperature on zooplankton in the alpine lakes.

#### UVR × nutrients

In a field study using mesocosms, Carrillo et al. (2008a) demonstrated that the interactive UVR × Nut effects on seston C:P ratio were antagonistic, i.e., the addition of nutrients ameliorated the effect of UVR decreasing C:P and hence improving food quality for herbivores (see reference 37 in Figure S6). Villar-Argaiz et al. (2012) subsequently tested the effects of these two stressors on the growth of three species of zooplankton with contrasting life history traits in field-coupled bioassays. Their results, that allowed discrimination between food quality and food quantity, showed that interactive effects of UVR × Nut on algal C:P ratio were antagonistic, i.e., the addition of nutrients dampened the detrimental effects of UVR on the growth of herbivorous zooplankton (Figure 5C).

#### Light × nutrients

Dickman et al. (2008) manipulated light and nutrients to test the general hypothesis that food chain efficiency (and hence herbivore production) was constrained by the nutritional quality of the food. The combined effect of light and nutrients yielded synergistic effects on seston C:N and C:P ratios in treatments without fish (see reference 6 in Figure S1), which resulted in increased food quality that favored the production of zooplankton (Figure 5D). In a similar study, Plum et al. (2015) found that the nature of the interactive effects of light and nutrients on algal C:P and N:P ratios were mostly synergistic (four out of five algal bioassays; see reference 20 in Figure S1), and it was under high light and N reduced conditions when the highest copepod biomass was attained. Further, in a recent study, Rock et al. (2016) identified that carnivores can affect the mediated effects of light and nutrients on aquatic food chain efficiency. Interestingly, if we examine their carnivore-bluegill treatment (see reference 25 in Figure S1), the effects of light and nutrient on seston C:P were synergistic, and under these conditions herbivore efficiency was at its highest. Our analysis of the above studies suggest that synergistic effects of L × Nut resulted in enhanced food quality (decreased autotroph C:P) for zooplankton herbivores (Figure 5D).

From the heterogeneous paired-stressor studies analyzed above, the idea emerges that the identity of the stressor might mediate on the nature of the interaction with a second stressor, and in turn on herbivore performance. Thus, the harmful UVR antagonistic interaction with nutrients, and the synergistic interaction of light with nutrients on autotrophs, could both benefit zooplankton by decreasing C:P ratios in their food resources (Figures 5C,D). Altogether, these studies strongly highlight the importance of considering both the direction and magnitude of interactive impacts when evaluating ecological interactions if we are to advance in the theory of food chain in nature.

## Discussion and conclusion

Across all stressor pairs considered in the present study the following patterns emerged: (1) Antagonisms were the prevalent interaction in freshwaters, both streams and lakes, (2) both synergisms and antagonisms were co-dominant in marine systems with synergisms being slightly more common, and (3) overall non-additive effects were the dominant interactions in all aquatic ecosystems studied.

While the lack of data impeded comparisons among all of the paired stressors, we found pronounced system-specific differences. For example, with regards to the L × Nut interactions, our data for C:P and N:P is consistent with the initial hypothesis that the interaction was primarily synergistic in the oceans, but not in freshwater systems where interactions were more evenly distributed between synergism and antagonism. As for C:N, the present study showed no differences between stream and lakes, but a sharp contrast between freshwater systems and the ocean. We suggest that this could possibly be associated with differences in the nutrient limiting freshwater and marine systems (e.g., Moore et al., 2013). The inherent similarities that we find for freshwater systems are possibly due to the similar stoichiometric principles that apply in “green” autotroph-based food webs and the “brown” detritus-based systems that dominante in lakes and streams, respectively (Evans-White and Halvorson, 2017).

While discussing the interactive effects of changing precipitation, decreasing DOC (“sun screen”), increasing UV-penetration, and continuing acid-rain deposition/mobilization from sediments in the Canadian Shield Lakes, Gorham (1996) made the following observation: “Most of our diverse impacts on the environment are studied as separate problems, and only rarely do scientists examine appropriately their many and complex physical, chemical and biological interactions.” As is now revealed by the analyses of interaction of stressors (Crain et al., 2008; Jackson et al., 2015, present study), non-additive responses predominate across aquatic ecosystems. Our study finds strong evidence that the nature of the interactive effect depends on the stressor pair at play, with a wide range of variability in the net effects of the interactions, with some observations supporting antagonism while others synergism.

Interesting questions arise from these observations regarding the basis for the commonalities as well as differences across ecosystems: Why are non-additive interactions prevalent across such a wide variety of aquatic ecosystems? Could the inherent heterogeneity of freshwater systems versus the relative uniformity of marine systems, and/or variability in the type and magnitude of stressor pairs, be responsible for the observed inter-ecosystem differences? How do we ascribe specific interaction effects of stressor pairs (e.g., light and nutrients on EOC-production in phytoplankton) with specific ecological phenomena (e.g., microbial growth and respiration)? Finally, how do we go about studying the interactive effects of all the stressors that are acting on an ecosystem at a given time?

Our findings highlight the importance of non-additive effects of interacting stressors on ecosystem processes and the need for further studies evaluating the interactive effects for developing a more rigorous comparative ecology. Serving as key feedback loops in ecosystems, the balance between these sensitive antagonistic and synergistic responses affects everything from small scale phenomena such as food chain efficiency to large scale phenomena such as ecosystem carbon and nutrient cycling (Schlesinger and Bernhardt, 2013). Of recent, there has been a resurgence of interest in role of multiple stressors on aquatic ecosystems (Allan et al., 2012). However, very few studies have explored the possibility of untangling the issue of interactions among these stressors (2 factor and more) on higher-order phenomena (Jackson et al., 2015; present study). The findings from our study suggesting how interactions of multiple stressors may result in subtle shifts in the net responses (synergistic or antagonistic) point to the importance of stressor interaction studies in the future. Today, ecologists are being challenged to predict dynamic tipping points of ecosystems shifting thresholds under a confluence of conditions where interactive effects play a key role (Scheffer et al., 2012; Duarte, 2014; Steffen et al., 2015; Costanza, 2017). As Odum (1964) pointed out decades ago, the challenging study of the emergent properties of ecosystems is the new ecology. If non-additive effects of interactions should be the norm in ecosystems, it now appears that an ecosystem is more than even the sum of its interactions. In a rapidly changing world, gaining a better understanding of how multiple stressors interact and the predictive modeling of their complex outcomes, should be an urgent interdisciplinary priority.

## Author contributions

MV-A, JM-S, and PC conceived the original idea for this study, with inputs from BB. MV-A, JM-S, BB, and PC wrote, approved and equally contributed to the final version of the manuscript.

### Conflict of interest statement

The authors declare that the research was conducted in the absence of any commercial or financial relationships that could be construed as a potential conflict of interest.
