# Supplementary figures and images for: Predominant Non-additive Effects of Multiple Stressors on Autotroph C:N:P Ratios Propagate in Freshwater and Marine Food Webs
Source: Front Microbiol. 2018 Jan 30;9:69. doi: 10.3389/fmicb.2018.00069 (PMC5797581; doi:10.3389/fmicb.2018.00069)

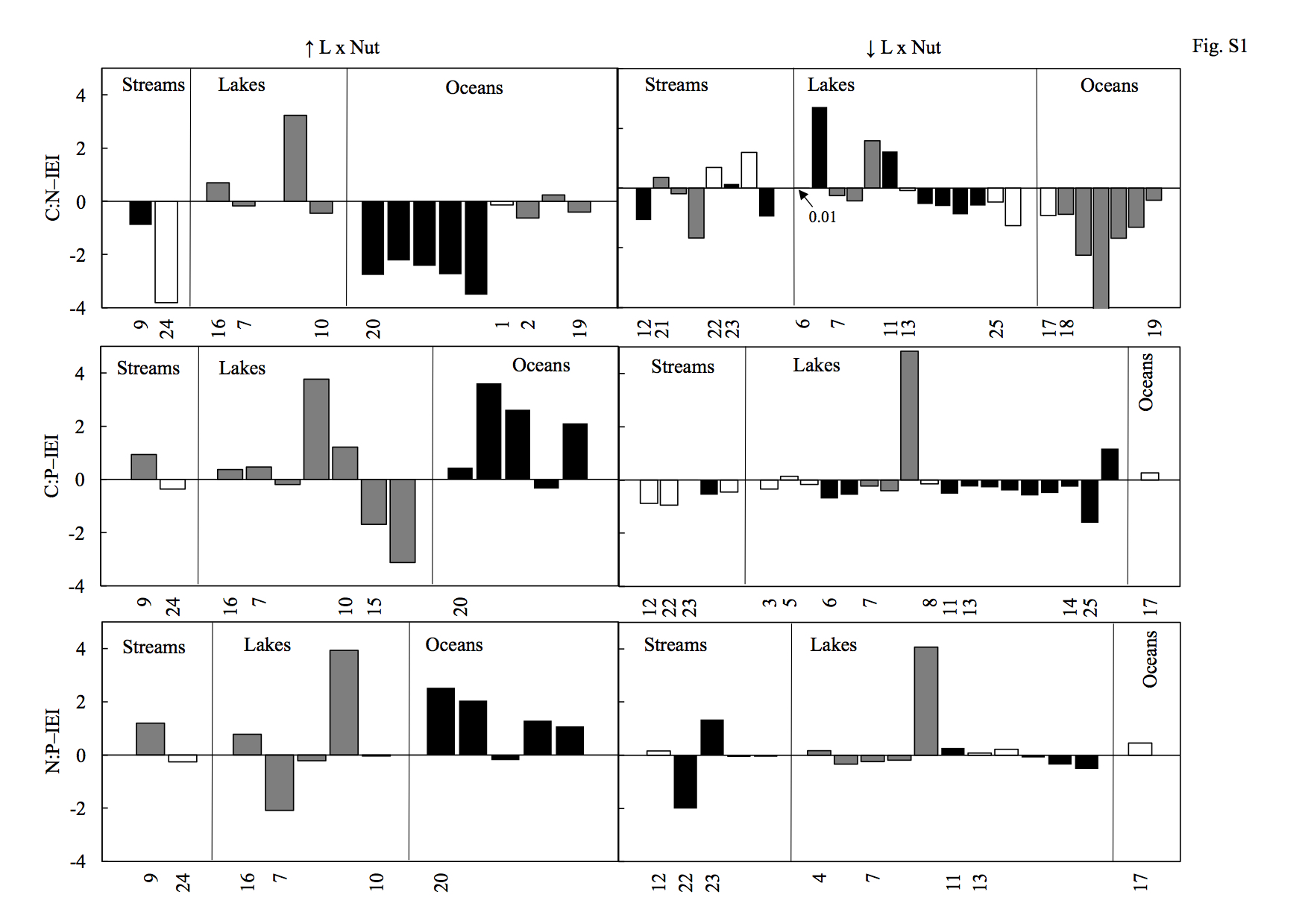

Supplement: Supplementary file 1 [file Image1.JPEG]

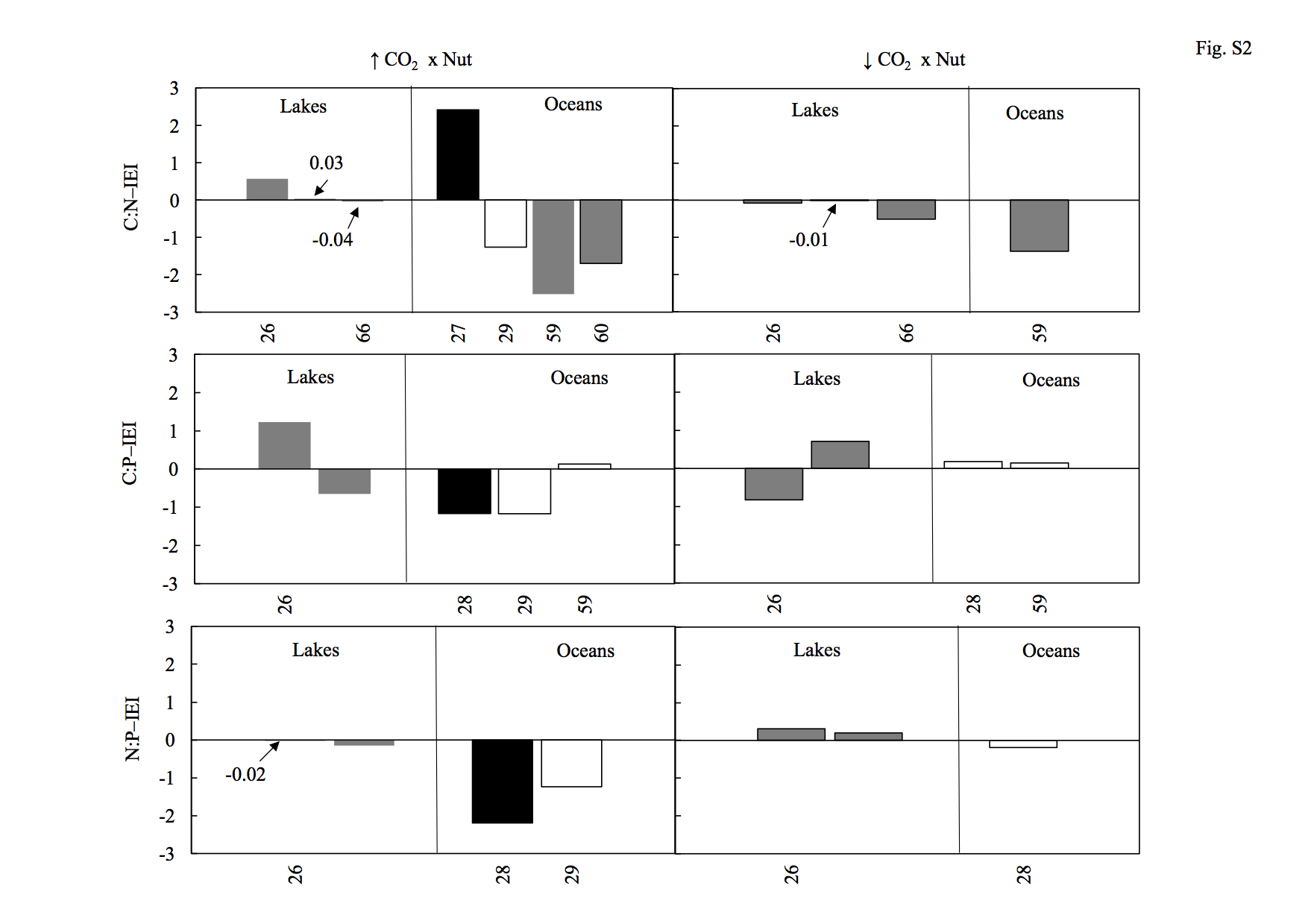

Supplement: Supplementary file 2 [file Image2.JPEG]

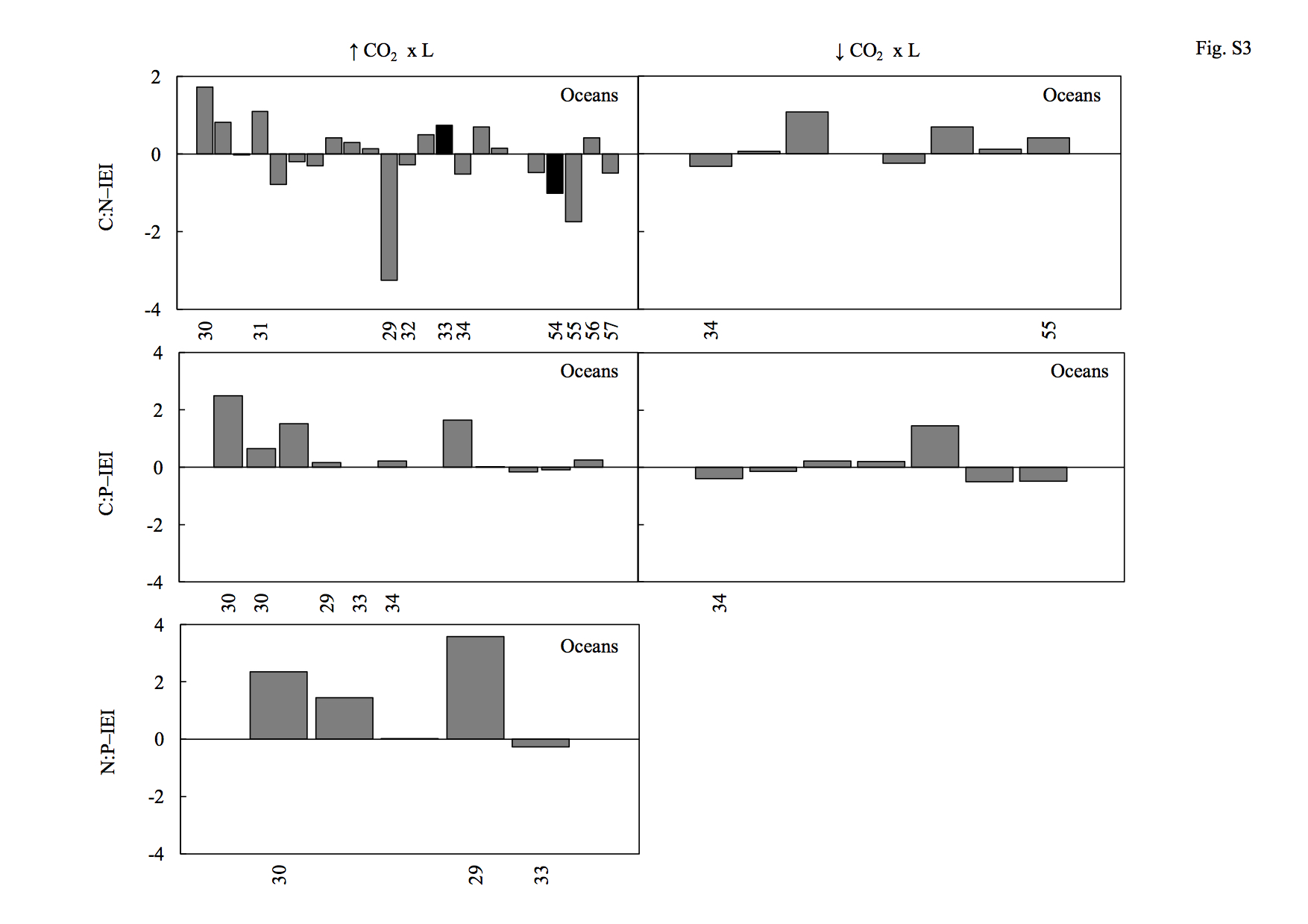

Supplement: Supplementary file 3 [file Image3.JPEG]

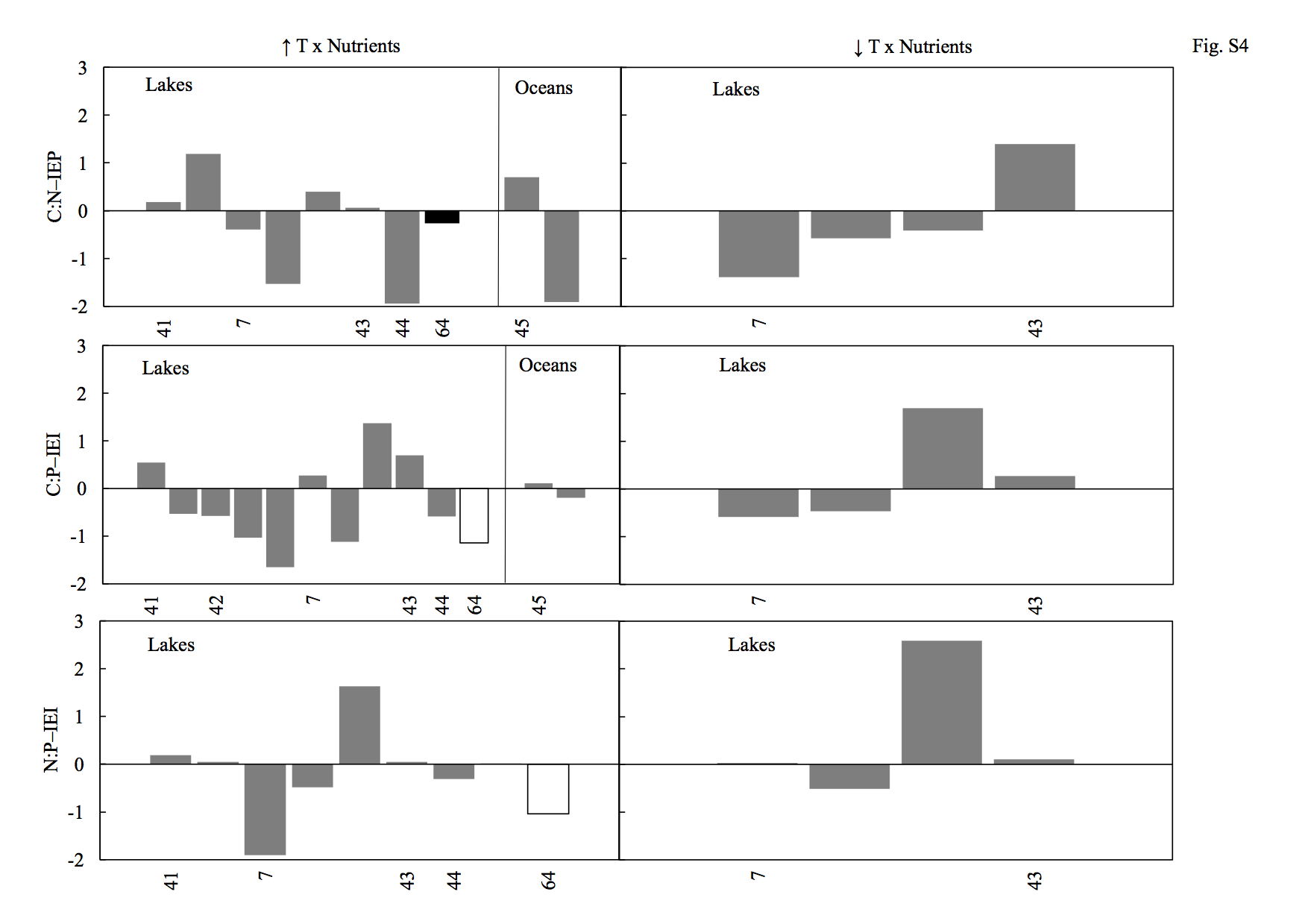

Supplement: Supplementary file 4 [file Image4.JPEG]

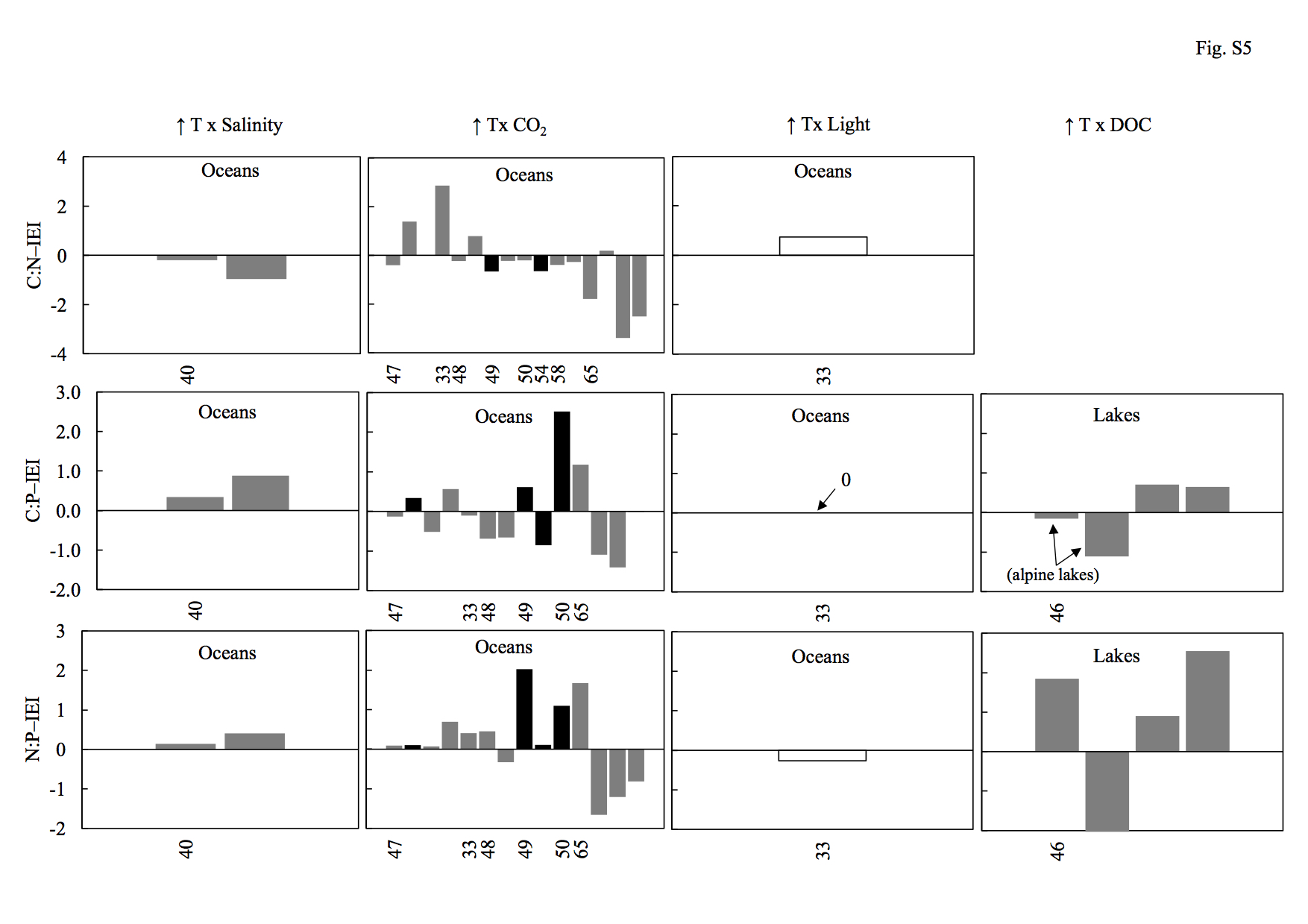

Supplement: Supplementary file 5 [file Image5.JPEG]

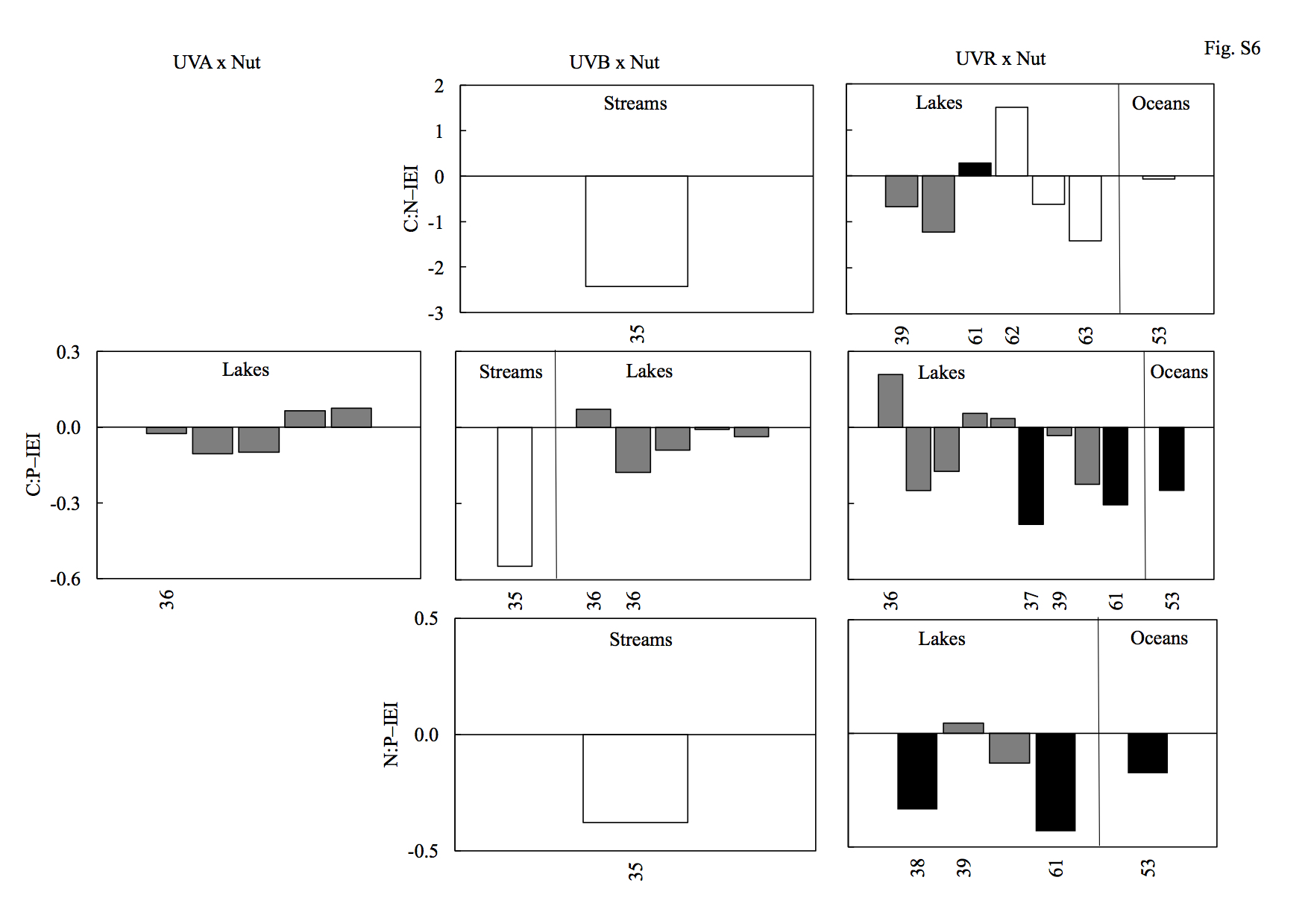

Supplement: Supplementary file 6 [file Image6.JPEG]
