# Supplementary material for: Predominant Non-additive Effects of Multiple Stressors on Autotroph C:N:P Ratios Propagate in Freshwater and Marine Food Webs
Source: Front Microbiol. 2018 Jan 30;9:69. doi: 10.3389/fmicb.2018.00069 (PMC5797581; doi:10.3389/fmicb.2018.00069)
Supplement: Supplementary file 7 [file Table1.DOCX]

**Table S1.** Summary of published studies on the effects of paired stressors on the C:N:P ratio of autotrophs.

**Column legend**

**# Ref.**: Number corresponds to the citation under Supplementary Reference and Figures S1-S6.

**Stressors**: Stressor pairs (CO_2_, carbon dioxide; Nut, Nutrients; L, light; T, temperature; DOC, dissolved organic carbon; UVR, ultraviolet radiation).

**Projection tested**: Scenarios tested in the original study for each of the two stressors. ↑ increase stressor; ↓ decrease stressor.

**Levels**: Stressor treatment levels tested in this study. In parenthesis are control vs. experimental treatments, respectively.

**Interactive effects**: Type of the interaction outcomes (A, antagonistic; S, synergistic; Ad, additive) and statistical significance (s, significant; ns, not significant; nt, not tested in the original study) found for the stressor pairs on C:N:P ratios. Given within parenthesis are: Type of interaction, significance, number of observations.

**Habitat**: Streams, Lakes, Oceans.

**Species/community**: Species or communities used in the original study. In parenthesis is the major group affiliation of taxa.

**Experiment type**: Laboratory (L) or field (F) experiment.

**Conditions:** Details on temperature, light (intensity, light:dark cycle, etc.), or nutrients in the original experiments.

| **# Ref.** | **Stressors** | **Projection tested** | **Levels** | **Interactive effects** | **Habitat** | **Species/community** | **Type experiment** | **Conditions** |
| --- | --- | --- | --- | --- | --- | --- | --- | --- |
| 27 | CO_2_ x N | CO_2_ ↑  ↓/↑ N | CO_2_  (390 vs. 1000 µatm)  Nitrogen  (control media vs. 100 µmmol N L^-1^ NO_3_^-^, control media vs. 10 µmmol N L^-1^ NO_3_^-^) | C:N (S, s, 1) | Oceans | *Phaeodactylum tricornutum* Bohlin CCMA 106 (diatom) | L: Semicontinuos batch cultures in exponential growth phase | 20ºC, 70 µmol photons m^-2^ s^-1^, 12:12 L/D cycle |
| 26 | CO_2_ x N | ↑/↓ CO_2_  ↑ N, P | *p*CO2 (400 vs. 550, and 400 vs. 250 *µ*atm)  Nutrients (5 vs. 25, and 5 vs. 100 µg L^-1^ P) | C:N (A/S, nt, 4)  C:P (A/S, nt, 4)  N:P (A/S, nt, 4) | Lakes | Natural phytoplankton assemblage | L: Semicontinuous experiment in 20-L aquatainers | 25°C, ~80 µmol photons m^-2^ s^-1^, 12:12 L/D cycle, Comparisons of 3 of 5 P treatments |
| 59 | CO_2_ x N | ↑/↓ CO_2_  ↑ P | *p*CO2 (400 vs. 730 ppm, and 400 vs. 220 ppm)  P (P-limited vs. P-replete; i.e. 0.5 vs. 20 µmol L^-1^) | C:N (A, nt, 2)  C:P (S, ns, 2) | Oceans | *Pseudo-nitzschia* (diatom) | L: Semicontinuous batch cultures in steady-state growth | 120 µmol photons m^-2^ s^-1^, 12:12 L/D cycle |
| 28 | CO_2_ x N | ↑/↓ CO_2_  ↑ Vit. B12 | CO_2_ (370 vs. 670 ppm, and 370 vs. 200 ppm)  Vitamin B12 (7 vs. 370 ppm) | C:P (A/S, s/ns, 4)  N:P (A, s or ns, 4) | Ocean | *Attheya* sp. (diatom) | L: Semicontinuous batch cultures until steady-state growth rate | 3ºC, 80 µmol photons m^-2^ s^-1^, 12:12 L/D cycle |
| 29 | CO_2_ x N | ↑ CO_2_  ↑ Fe | CO_2_ (380 vs. 750 ppm)  Fe (ambient vs. enriched Fe) (1nM FeCl_3_ in 0.01 N HCl) | C:N (A, ns, 1)  C:P (A, ns, 1)  N:P (A, ns, 1) | Ocean | Natural phytoplankton | F/L: Shipboard continuous incubation (`Ecostat´ system) | 13 days experiment at a dilution rate of 0.4 d^-1^ (0.2 µm filtered seawater) |
| 60 | CO_2_ x N | ↑ CO_2_  N source | CO2 (400 vs. 1000 ppm)  N-source (NH_4_^+^ + NO_3_^-^ vs. NO_3_^-^) | C:N (A, nt, 1) | Ocean | *Emiliania huxleyi*  (Coccolithophore) | L: Continuous cultures in steady-state growth | 17ºC, 5 months of continuous culturing |
| 66 | CO_2_ x N | ↑/↓ CO_2_  ↑ N | CO_2_ (400 vs. average at 800 and 2800 ppm, and 400 vs. average at 0.5, 50, 100 ppm)  N (low vs. high N; 0.4 mmol L-1 vs. 12.0 mmol L-1 NaNO3) | C:N (A, nt, 2) | Lakes | *Microcystis aeruginosa* CYA140 (cyanobacteria) | L: chemostats | 24°C, 50 *µ*mol photons m^-2^ s^-1^ |
| 30 | CO_2_ x L | ↑ CO_2_  ↑ L | CO_2_ (360 vs. 2000 ppm)  Light (80 vs. 500 µmol m^-2^ s^-1^) | C:N (A/S, nt, 3)  C:P (S, nt, 3)  N:P (S, nt, 3) | Ocean | *Emiliania huxleyi* (Lohmann) Hay & Mohler  (Coccolithophore) | L: Chemostats and batch cultures | 20ºC, 14:10 L/D cycle |
| 33 | CO_2_ x L | ↑ CO_2_  ↑ L | CO_2_ (375 vs. 750 ppm)  Light (50 and 400 µmol m^-2^ s^-1^) | C:N (S, s, 1)  C:P (Ad, ns, 1)  N:P (A, ns, 1) | Ocean | *Emiliania huxleyi* (coccolithophore) | L: Semicontinuous batch cultures at exponential growth phase | 50 µmol photons m^-2^ s^-1^, 12:12 L/D cycle |
| 34 | CO_2_ x L | ↓/↑ CO_2_  ↑ L | CO_2, aq_ (10-20 vs. < 10-20 mmol Kg^-1^)  Light (L:D cycle vs. continuous light) | C:N (A/S/Ad, nt, 14)  C:P (A/S, nt, 14) | Ocean | *Phaeodactylum tricornutum*  *Skeletonema costatum*  *Asterionella glacialis*  *Thalassiosira weissflogii*  *Thalassiosira punctigera*  *Coscinodiscus wailesii*  (diatoms)  *Scrippsiella trochoidea* (dinoflagellate) | L: Dilute batch cultures, CO_2, aq_  adjusted by the addition of HCl or NaOH | 15ºC, 150 µmol photonos m^-2^ s^-1^, 18:6 (*T. Weissflogii*) and 16:8 L/D cycle (other species) |
| 32 | CO_2_ x L | ↑ CO_2_  ↑ L | CO_2_ (400 vs. 800 µatm)  Light (30 vs. 250 µmol photons m^-2^ s^-1^) | C:N (A/S, nt, 2) | Ocean | *Thalassiosira pseudonana*  (diatom) | L: Semi-continuous cultures at exponential phase of growth | Continuous light, NO_3_^-^ or NH_4_^+^ as nitrogen source |
| 54 | CO_2_ x L | ↑ CO_2_  ↑ L | CO_2_ (ambient vs. future pCO2)  Light (35 vs. 65 µmol photons m^-2^ s^-1^) | C:N (A, s, 1) | Ocean | *Thalassiosira weissflogii* Grunnow CCMP 1053  (diatom) | L: Semicontinuous cultures at constant temperature of 20ºC | saturating irradiance ≥135 μmol photons m^−2^ s^−1^ |
| 55 | CO_2_ x L | ↑/↓ CO_2_  ↑ L | CO_2_ (200-246 vs. 506-599 µatm, and 200-246 vs. 135-152 µatm)  Light (40 vs. 240 µmol photons m^-2^ s^-1^) | C:N (A/S, nt, 2) | Ocean | *Proboscia alata* Brightwell  (diatom) | L: Semicontinuous bath cultures (6 L polymethylmetacrylate vessels) | 3ºC, 16:8 L/D cycle |
| 56 | CO_2_ x L | ↑ CO_2_  ↑ L | pCO_2_ (390 vs. 750 ppmv)  Light (30 vs. 380 µmol photons m^-2^ s^-1^) | C:N (S, nt, 1) | Ocean | *Thalassiosira pseudonana* CCMP 1335  (diatom) | L: Bioreactors in batch and turbidostat modes | Comparison of 2 out of 5 light treatments |
| 57 | CO_2_ x L | ↑ CO_2_  ↑ Light | CO_2_ (150 vs. 900 µatm)  Light (50 vs. 200 µmol photons m^-2^ s^-1^) | C:N (A, nt, 1) | Ocean | *Trichodesmium erythraeum* (IMS101)  (Cyanophycea) | L: Bath cultures in midexponential growth phase (1-L cylindrical glass flasks) | 25ºC, 12:12 L/D cycle |
| 31 | CO_2_ x L | ↓ CO_2_  ↓/↑ L | CO_2_ (380 vs. 180 µatm)  Light (200 vs. 500, and 200 vs. 20 µmol photons m^-2^ s^-1^) | C:N (A/S, nt, 7) | Ocean | *Fragilariopsis curta*  *Odontella weisflogii*  (diatoms) | L: Semicontinuous cultures | 4ºC, 16:8 L/D cycle |
| 29 | CO_2_ x L | ↑ CO_2_  ↓ L | CO_2_ (380 vs. 750 ppm)  Light (~33 vs. 7% incident sea surface light) | C:N (A, ns, 1)  C:P (S, ns, 1)  N:P (S, ns, 1) | Ocean | Natural phytoplankton | Shipboard continuous incubation (`Ecostat´ system) | 13 days experiment at a dilution rate of 0.4 d^-1^ (0.2 µm filtered seawater) |
| 2 | L x Nut | ↑ L  ↓ N/P | Light (50 vs. 600 µmol photons m^-2^ s^-1^)  N and P limitation (NO_3_^-^:PO_4_^3-^ ratios of 5 vs. 35 mol N mol P^-1^) | C:N (A/S, nt, 2) | Ocean | *Chaetoceros muelleri* (Bacillariophyceae) | L: Batch cultures, cells at early and late exponential growth phases | 25ºC |
| 1 | L x Nut | ↑ L  ↑ Fe | Light (25 vs. 100 µmol photons m^-2^ s^-1^)  Fe (Starved vs. replete, 1 µM FeCl3) | C:N (A, ns, 1) | Ocean | *Chaetoceros brevis* (Bacillariophyceae) | L: Batch cultures | 4ºC, 8:16 L/D cycle |
| 7 | L x Nut | ↑ L  ↓ N, P | Light (130 vs. 450 µmol photons m^-2^ s^-1^)  N, P (no limitation vs. N or P limitation) | C:N (A/S, nt, 2)  C:P (A/S, nt, 3)  N:P (A/S, nt, 3) | Lakes | *Chaetoceros wighamii*  (Bacillariophyceae) | L: Batch culture at exponential and stationary growth phases | 7ºC, 16:8 L/D cycle |
| 24 | L x Nut | ↑ L  ↑ N, P | Light (5 vs. 250 µmol photons m^-2^ s^-1^)  NP (ambient vs. enriched, i.e. 180 µmol N L^-1^, 4 µmol P L^-1^) | C:N (A, ns, 1)  C:P (A, ns, 1)  N:P (A, ns, 1) | Streams | Periphyton | L: 1-L aquaria, comparisons of treatments with no grazers | 20ºC, 20:4 L/D cycle |
| 16 | L x Nut | ↑ L  ↓ P | Light (10 vs. 70 µmol quanta m^-2^ s^-1^)  P (moderate vs. severe P limitation; i.e. 10 vs. 1 µmol P L^-1^) | C:N (S, nt, 1)  C:P (S, nt, 1)  N:P (S, nt, 1) | Lakes | *Selanastrum capricornutuum* | L: Continuous culture systems | No Nut limitation at 50 µmol P L^-1^ and increasing degree of P limitation at ≤10 µmol P L^-1^ |
| 9 | L x Nut | ↑ L  ↑ N, P | Light (control vs. 2000 watt)  N, P (ambient vs. 10X ambient) | C:N (A, s, 1)  C:P (S, nt, 1)  N:P (S, nt, 1) | Streams | Benthic algal community | F/L: Flow-through channels, only ungrazed treatments compared | N and P to 210 µg N/1 as NaNO_3_, 40µg N L^-1^ as NH_4_Cl, and 35 µg P L^-1^ as K_2_HPO_4_, |
| 10 | L x Nut | ↑ L  ↑ P | Light (10 vs. 70 µmol m^-2^ s^-1^)  P (1 vs. 50 µm P L^-1^) | C:N (A, nt, 1)  C:P (S, nt, 1)  N:P (A, nt, 1) | Lakes | *Selenastrum captricornutum*  (green algae) | L: Chemostat cultures, comparions are average for six sample days | Values compared from 2 of 7 P treatments |
| 15 | L x Nut | ↑ L  ↑ P | Light (12 vs. 260 µE m^-2^ s^-1^)  P (0.1 vs. 1.6 µM P, and 0.1 vs. 10 µM P) | C:P (A, nt, 2) | Lakes | *Scenedesmus acutus*  (green algae) | L: Semibatch cultures | N/P ratio held at 80:1 (molar), Values compared from 3 of 6 nutrient treatments |
| 20 | L x Nut | ↑ L  ↑ N | Light (30 vs. 120 µE m^-2^ s^-1^)  N (control media vs. 1080 µmol N L^-1^) | C:N (A, s, 5)  C:P (A/S, s, 5) N:P (A/S, s, 5) | Ocean | *Rhodomonas salina* (cryptophyte)  *Tetraselmis* sp. (prasinophyte)  *Skeletonema costatum*  (diatom)  *Chaetoceros danicus* (diatom)  Mixture of 4 above species | L: Batch cultures | 18 ºC, 12:12 L/D cycle |
| 19 | L x Nut | ↑ L  N source | Light (61 vs. 198, and 61 vs. 5 µE m^-2^ s^-1^)  N enrichment (control media vs. 75 µM NO_3_^-^ vs. NH_4_^+^) | C:N (A, nt, 2) | Ocean | *Thalassiosira pseudonana*  (diatoms) | L: Cultures in 50 ml glass test tubes | 17.5ºC, continuous light, cultures harvasted at mid-exponential phase |
| 7 | L x Nut | ↓ L  ↓ N, P | Light (130 vs. 40 µmol photons m^-2^ s^-1^)  Nutrient (no limitation vs. N or P limitation) | C:N (A/S, nt, 3)  C:P (A/S, nt, 3)  N:P (A/S, nt, 3) | Lakes | *Chaetoceros wighamii*  (Bacillariophyceae) | Batch culture at exponential and stationary growth phases | 7ºC, 16:8 L/D cycle |
| 13 | L x Nut | ↓ L  ↑ N, P | Light (average for mixed layer vs. lowest supply as measured in the field)  Nutrients (ambient vs. NP, 75 µmol L^-1^ N and 7 µmol L^-1^ P) | C:N (A, s/ns, 5)  C:P (A, s, 5)  N:P (S/A, s/ns, 5) | Lakes | Natural phytoplankton | L: Five experiments using flasks with natural phytoplankton, comparisons of light treatments between light measured at average mixed layers vs. lowest light measured in the field | 25ºC, Light intensity (PAR) ranged 8-600 µmol m^-2^ s^-1^ |
| 6 | L x Nut | ↓ L  ↑ N, P | Light (ambient vs. 90% reduction)  Nutrient (ambient vs. high Nut, 50 µg N L^-1^and 5 µg P L^-1^) | C:N (A, s, 2)  C:P (A, s, 2) | Lakes | Natural seston | F: 5000-L mesocosm, in situ incubations | Treatments with and withour fish, nutrients added 3 times per week |
| 11 | L x Nut | ↓ Light  ↑ N, P | Light (ambient vs. 10% ambient light; 550 vs. ~50 µmol m^-2^ s^-1^)  N, P (ambient vs. N and P) amendments of 50 µg N L^-1^ and 5 µg P L^-1^ | C:N (S, s, 1)  C:P (A, s, 1)  N:P (S, s, 1) | Lakes | Natural phytoplankton | F: 5000-L mesocosms | Comparisons among treatments without fish, nutrients added 3 times/week |
| 12 | L x Nut | ↓ Light  ↑ N, P | Light (ambient and 65% reduction)  N, P (ambient vs. very high N and P); Nitrate (91 vs. 933 µg L^-1^), Phosphate (14-141 µg L^-1^) | C:N (A, s, 1)  C:P (A, ns, 1)  N:P (S, ns, 1) | Streams | Stream diatoms | F: Circular stream channels | Comparisons of treatments without grazers |
| 3 | L x Nut | ↓ Light  ↑ N, P | Light (ambient and 90% reduction)  N and P supply (low ambient vs. 10 x supply) | C:P (A, ns, 1) | Lakes | Natural seston | F/L: Mesocosms  (300-L cattle tanks) | Mesocosms inoculated with natural assemblages of algae and zooplankton |
| 4 | L x Nut | ↓ Light  ↑ N, P | Light (ambient and 90% reduction)  N and P supply (low supply vs. 10xlow supply) | N:P (S/A, nt, 2) | Lakes | Natural seston | Mesocosms  (300-L cattle tanks), inoculated with natural assemblages of algae and zooplankton | Three N:P ratios (4, 14 and 50) |
| 5 | L x Nut | ↓ Light  ↑ N, P | Light (100 vs 50% ambient light, and 100 vs. 25% ambient light)  N, P (ambient vs. NP addition, P (5, 10, 20 µmol P m^-2^ d^-1^ and N to N:P ratio of 75 | C:P (A/S, ns, 2) | Lakes | Natural seston | F: Mesocosms (polyethylene tubes of 25 m^3^) | Ambient *in situ* conditions |
| 14 | L x Nut | ↓ Light  ↑ N, P | Light (100 vs. 7% ambient light)  N, P (No NP vs. 0.75, 1.5, 3, 6 and 12 µg P L^-1^ and N to an N:P ratio of 30) | C:P (A, s, 1) | Lakes | Natural phytoplankton | F: 3.1 m^3^ *in situ* enclosures, values compared between control vs. most enriched treatment | Mean PAR at surface (35 moles photons m^-2^ d^-1^, range 18-48), |
| 21 | L x Nut | ↓ Light  ↑ N, P | Light (100 vs. 10% ambient light)  N, P (ambient vs. 0.45 g N and 0.11 g P m^-2^ d^-1^) | C:N (A/S, nt, 3) | Streams | Periphyton | F: *In situ* experiments in natural creeks | Fertilization treatments, Shade cloth with 90% shading effects |
| 22 | L x Nut | ↓ Light  ↑ N, P | Light (incubation at 0.4 vs. 1.4 m depth; ~400 vs. ~40 µW/cm^2^)  N, P (ambient vs. 50 µM N and 10 µM P) | C:N (S, ns, 1)  C:P (A, ns, 1)  N:P (A, s, 1) | Streams | Periphyton | F: Cylindrical transparent plastic containers (2 m diameter, 2.4 m tall) | Periphyton substrates (0.04 m^2^ plastic tiles) |
| 23 | L x Nut | ↓ Light  ↑ N, ↑ P, ↑ N, P | Light (irradiance at near 10 vs. 200 cm depth; ~3% vs ~80% reduction incident light)  N, P (ambient vs. +N, +P, +NP; 0.5 M NaNO_3_, 0.05 M NaH_2_PO_4_, 0.5 NaNO_3_ + 0.05 M NaH_2_PO_4_) | C:N (A/S, s, 3)  C:P (A/S, s/ns, 3)  N:P (A/S, s, 3) | Streams | Periphyton | F: *In situ* nutrient-diffusing substrata (NDS) | periphyton colonization of a small piece of plankton net |
| 25 | L x Nut | ↓ Light  ↑ N, P | Light (65 vs. 10% ambient light)  N, P (ambient vs. high NP; 50 µgN L^-1^ and 5 µg P L^-1^ 3 x per week) | C:N (A, ns, 2)  C:P (A/S, s, 2) | Lakes | Natural phytoplankton assemblage | F: Polyethylene 5000-L mesocosms,  mesocosms with *Chaoborus* or bluegills | All mesocosms received and initial large pulse of nutrients 150 µg N L^-1^ and 15 µg P L^-1^ |
| 8 | L x Nut | ↓ Light  ↑ P | Light (100 vs. 25% ambient light)  P (ambient P vs. 0.001, 0.005 and 0.010 mol P L^-1^) | C:P (A, ns, 1) | Lakes | Epilithon | F: Nutrient-diffusing substratum experiments using inverted clay pots | Ambient *in situ c*onditions, N (NH_4_NO_3_) added to mantain N:P in the agar >7 |
| 17 | L x Nut | ↓ Light  ↑ Fe | Light (high vs. low light, 33 vs. 7% sea surface irradiance)  Fe (ambient vs. 1 nM Fe Cl_3_) | C:N (A, ns, 1)  C:P (S, ns, 1)  N:P (S, ns, 1) | Ocean | Natural phytoplankton assemblage | F/L: Shipboard continuous incubation using a an ‘‘Ecostat’’ system | Fe (1nM FeCl_3_ in 0.01 N HCl) |
| 18 | L x Nut | ↓ Light  ↑ Fe | Light (high vs. low light, 45 vs. 15% sea surface irradiance)  Fe (ambient vs. 2 nM acidified FeCl_3_) | C:N (A, nt, 5) | Ocean | Natural phytoplankton assemblage | F/L: On deck incubations of 2.1 L bottles | Five experiments (5-6 d incubations) |
| 40 | T x Sal | ↑ T  ↓ Sal | T (7 vs. 15 ºC, and 7 vs. 24ºC)  Salinity (10, 34‰) | C:N (A, nt, 2)  C:P (S, nt, 2)  N:P (S, nt, 2) | Lakes | *Gymnodinium galatheanum* Braarud  (dinoflagellate) | L: Batch cultures in exponential growth phase | 255 µmol m^-2^ s^-1^, 18:4 L/D cycle |
| 46 | T x DOC | ↑ T  ↑ DOC | T (10 vs. 17ºC)  DOC (ambient vs. ambient+80%) | C:P (A, s/nt, 4)  N:P (A/S, nt, 4) | Lakes | Natural phytoplankton assemblage | L: 8-L transparent, cylindrical PVC Nalgene bottles in growth chambers | Experiments in two alpine and two montane lakes |
| 42 | T x Nut | ↑ T  ↑ P | T (12 vs. 16ºC, 12 vs. 20ºC, 12 vs. 24ºC)  P supply levels (0.25 vs. 2 µM) | C:P (A, nt, 3) | Lakes | *Scenedesmus obliquus*  (green algae) | L: Semi-batch cultures at four differnt P supply levels (0.25, 0.5, 1 and 2 µM) | 50 µmol quanta m^-2^ s^-1^, comparisons betwen 2 of 4 P supply levels |
| 41 | T x Nut | ↑ T  ↑ N | T (4 vs. 8 ºC, and 4 vs. 12ºC)  N (N-deficient vs. N-replete; N:P = 9 vs. N:P = 30) | C:N (S, nt, 2)  C:P (A/S, nt, 2)  N:P (S, nt, 2) | Lakes | *Skeletonema costatum*  CCMP 1332 | L: 25-L polycarbonate bottles in a temperature-controlled chambers | Maximum light of 377 µmol photons m^-2^ s^-1^, 12:12 L/D cycle |
| 7 | T x Nut | ↑ T  ↓ N, P | T (7 vs. 11ºC )  N, P (no limitation vs. N, P or Si limitation) | C:N (A or S, nt, 3)  C:P (A or S, nt, 3)  N:P (A or S, nt, 3) | Lakes | *Chaetoceros wighamii*  (Bacillariophyceae) | L: Batch culture at exponential and stationary growth phases | 130 µmol photons m^-2^ s^-1^), 16:8 L/D cycle |
| 44 | T x Nut | ↑ T  ↑ N, ↑ P | T (~10 vs. ~20ºC)  N, P (N-limited vs. N-sufficient, 42 vs. 300 µM N; and P-limited vs. P-sufficient, 2 vs. 50 µM P) | C:N (A, nt, 1)  C:P (A, nt, 1)  N:P (A/S, nt, 2) | Lakes | *Scenedesmus* sp.  (green algae)  *Asterionella formosa*  (diatom) | L: Chemostat cultures at steady state | Continuous ilumination of 17.1 W m^-2^ |
| 45 | T x Nut | ↑ T  ↑ N, ↑ P | Tª (12 vs. 18ºC, and 12 vs. 24ºC)  N, P (low vs. high N, 16:8 vs. 128:2 N:P µmol L^-1^); and (low vs. high P, 32:2 vs. 16:8 N:P µmol L^-1^) | C:N (S/A, nt, 2)  C:P (S/A, nt, 2) | Lakes | Phytoplankton mixture of several species | L: Phytoplankton batch cultures (255 ml Erlenmeyer flasks) | 60 µmol photons m^-2^ s^-1^, 12:12 L/D cycle |
| 64 | T x Nut | ↑ T  ↑ N, P | T (unheated vs. heated at IPCC climate scenarios of ‘A2 + 50%’)  N, P (no NP vs. weeky additions of 54 mg P and 538 mg N) | C:N (A, s, 1)  C:P (A, ns, 1)  N:P (A, ns, 1) | Lakes | Natural plankton community | F/L: Outdoor experiment in 2800-L groundwater fed flow-through mesocosms |  |
| 7 | T x Nut | ↓ T  ↑ N, P | T (7 vs. 3ºC )  N, P (no limitation vs. N or P limitation) | C:N (A, nt, 3)  C:P (A/S, nt, 3)  N:P (A/S, nt, 3) | Lakes | *Chaetoceros wighamii*  (Bacillariophyceae) | L: Batch culture at exponential and stationary growth phases | 130 µmol photons m^-2^ s^-1^, 16:8 L/D cycle |
| 43 | T x Nut | ↑/↓ T  ↑ P | T (9-16 vs. 9-21ºC, and 9-16 vs. 9-11ºC)  P (1 vs. 100 µmol L^-1^) | C:N (S, ns, 2)  C:P (S, ns, 2)  N:P (S, ns, 2) | Lakes | Natural phytoplankton assemblage | F/L: Phytoplankton natural assemblages cultured in 10-L polycarbotante carboys for 48 days | 120 µmol photons m^-2^ s^-1^, comparisons between low and high P levels |
| 47 | T x CO_2_ | ↑ T  ↑ CO_2_ | T (25 vs. 29ºC )  CO_2_ (~39 vs. 76 Pa, i.e. present-day vs. projected year 2100 pCO_2_) | C:N (A/SAd, nt, 4)  C:P (A/S, nt, 4)  N:P (S, nt, 4) | Ocean | *Trichodesmium erythraeum*  (Cyanobacteria) | L: Semicontinuos batch cultures in steady-state growth phase | Cultures of Pacific and Atlantic Ocean at P-sufficient (20 µmol L^-1^) and P-limiting conditions (0.2 µmol L^-1^ phosphate) |
| 33 | T x CO_2_ | ↑ T  ↑ CO_2_ | T (20 vs. 24ºC)  CO_2_ (375 vs. 750 ppm) | C:N (S, ns, 1)  C:P (A, ns, 1)  N:P (S, ns, 1) | Ocean | *Emiliania huxleyi* (coccolithophore) | L: Semicontinuous batch cultures at exponential growth phase | 50 µmol photons m^-2^ s^-1^, 12:12 L/D cycle |
| 48 | T x CO_2_ | ↑ T  ↑ CO_2_ | T (20 vs. 24ºC)  CO_2_ (380 vs. 750 ppm) | C:N (A/S, nt, 2)  C:P (A, nt, 2)  N:P (A/S, nt, 2) | Ocean | *Synechococcus*  *Prochlorococcus*  (cyanobacteria) | L: Semicontinuous batch cultures at steady-state growth | 45 µmol photons m^-2^ s^-1^, 12:12 L/D cycle |
| 49 | T x CO_2_ | ↑ T  ↑ CO_2_ | T (20 vs. 24ºC)  CO_2_ (375 vs. 750 ppm) | C:N (A, s/ns, 2)  C:P (A/S, s, 2)  N:P (S, s, 2) | Ocean | *Heterosigma akashiwo*  (raphidophyte)  *Prorocentrum minimum*  (dinoflagellate) | L: Semicontinuos batch cultures | 100 µmol quanta m^-2^ s^-1^, 12:12 L/D cycle |
| 50 | T x CO_2_ | ↑ T  ↑ CO_2_ | T (25 vs. 31ºC)  *p*CO_2_ (400 vs. 900 µatm) | C:N (A, nt, 1)  C:P (S, nt, 1)  N:P (S, nt, 1) | Ocean | *Trichodesmium* IMS106 | L: Diluted batch cultures | ~80 µmol photons m^-2^ s^-1^, 12:12 L:D cycle |
| 54 | T x CO_2_ | ↑ T  ↑ CO_2_ | T (15 vs. 20ºC)  pCO_2_ (pH 8-8.6 vs. 7.5-8.3) | C:N (A, s, 1) | Ocean | *Thalassiosira weissflogii* Grunnow  CCMP 1053  (diatom) | L: Semicontinuous cultures, carbonate system adjusted using HCl, NaHCO_3_, and Na_2_CO_3_ | 35 μmol photons m^−2^ s^−1^, 12:12 L/D cycle |
| 58 | T x CO_2_ | ↑ T  ↑ CO_2_ | T (15 vs. 20ºC)  CO_2_ (400 vs. 1000 µatm) | C:N (A, nt, 2) |  | *Thalassiosira weissflogii* CCMP 1339  *Dactyliosolen fragilissimus* RCC3389 | Incubation vessels of 20-L polyethylene bags | Comparions when cultures reached N-depletion,  100 µmol m^-2^ s^-1^, 14:10 L/D cycle |
| 65 | T x CO_2_ | ↑ T  ↑ CO_2_ | T (16.5 vs. 22.5°C, cold vs. warm)  CO_2_ (500 vs. 3000 µatm) | C:N (A/S, nt, 4)  C:P (A/S, nt, 4)  N:P (A/S, nt, 4) | Ocean | Natural phytoplankton assemblage | L: 1400 L-mesocom experiment in temperature-controlled culture rooms  Bloom and post-bloom measures | Maximum light 383 µmol photons m^-2^ s^-1^, ~14:10 L/D cycle |
| 33 | T x L | ↑ T  ↑ Light | T (20 vs. 24ºC)  Light (50 vs. 400 µmol m^-2^ s^-1^) | C:N (S, ns, 1)  C:P (Ad, ns, 1)  N:P (A, ns, 1) | Ocean | *Emiliania huxleyi* (coccolithophore) | L: Semicontinuous batch cultures at exponential growth phase | 20ºC, 12:12 L/D cycle |
| 52 | Nutrient x supplied ratio | ↑ Nutrient  ↑/↓ N:P ratio | Nutrient (low vs. high concentration of N)  Supply N:P ratios (4, 13 and 50) | C:N (S, ns, 2)  C:P (A, s, 2)  N:P ( A/S, s, 2) | Streams | Periphyton | F/L: Once-through flumes (2.8 m long x 10 cm wide x 7 cm high vinyl gutters) | Nutrient (NaNO_3_ and NaH_2_PO_4_ · H_2_O) added at a continuos rate of ~0.8 ml min^-1^ |
| 37 | UVR x Nut | Ambient UVR  ↑ P | UVR (Full light vs. PAR)  P (ambient vs. 20, 30, 40 and 60 µgP L^-1^) | C:P (A, s, 1) | Lakes | Natural seston | F: 2.7 m^3^ mesocosms, N (NH_4_NO_3_) added to give a final N:P molar ratio of 30 | Comparisons between ambient vs. most enriched treatment |
| 38 | UVR x Nut | Ambient UVR  ↑ P | UVR (Full light vs. PAR)  P (ambient vs. 20, 30, 40 and 60 µgP L^-1^) | N:P (A, s, 1) | Lakes | Natural seston | F: 2.7 m^3^ mesocosms, N (NH_4_NO_3_) added to give a final N:P molar ratio of 30 | Comparison between ambient vs. most enriched treatments, |
| 36 | UVR x Nut | Ambient UVR  ↑ P | UVR (Full light vs. PAR)  UVB (full light vs. PAR+UVA)  UVA (PAR+UVA vs. PAR)  P (ambient vs. 60-200 µg P L^-1^) | C:P (A/S, nt, 15) | Lakes | Natural seston | F: *In situ* experiment using whirlpak bags | Comparison between ambient and highest P treatment; five experiments |
| 39 | UVR x Nut | Ambient UVR  ↑ P | UVR (UVR vs. PAR)  P (0.5 vs. 1 µm P L^-1^, and 0.5 vs. 5 µm P L^-1^) | C:N (A, nt, 2)  C:P(A, nt, 2)  N:P (A/S, nt, 2) | Lakes | *Selenastrum captricornutum* | L: Semicontinuous cultures | 18ºC, 70 µmol quanta m^-2^ s^-1^, 16:8 L/D cycle |
| 35 | UVR x Nut | Ambient UVR  ↑ DOM | UVB (Full light vs. Full light -UVB)  DOM (ambient vs. plus-DOM) | C:N (A, ns, 1)  C:P (A, ns, 1)  N:P (A, ns, 1) | Streams | Natural periphyton | F/L: Artificial streams | Natural DOM collected with a reverse osmosis system |
| 53 | UVR x Nut | Ambient UVR  ↑ P | UVR (Full light vs. PAR)  P (ambient vs. 30μg P L^-1^) | C:N (A, ns, 1)  C:P (A, ns, 1)  N:P (A, ns, 1) | Oceans | Natural phytoplankton | F/L: 20-L low-density polyethylene microcosm on tanks on deck of the boat | 6-d incubations |
| 61 | UVR x Nut | Ambient UVR  ↑ N, P | UVR (UVR+PAR vs. PAR)  N, P (ambient vs. 20 μg P L^−1^ and 50 μg N L^−1^) | C:N (S, s, 1)  C:P (A, ns, 1)  N:P (A, s, 1) | Lakes | Natural phytoplankton | F: *In situ* experiments of 10-L containers | 6-d incubations |
