# Supplementary material for: Predominant Non-additive Effects of Multiple Stressors on Autotroph C:N:P Ratios Propagate in Freshwater and Marine Food Webs
Source: Front Microbiol. 2018 Jan 30;9:69. doi: 10.3389/fmicb.2018.00069 (PMC5797581; doi:10.3389/fmicb.2018.00069)
Supplement: Supplementary file 8 [file Table2.DOCX]

**References cited in Table S1 and Figs S1-S6**

1. Van Oijen T, Van Leeuwe MA, Gieskes WWC, de Baar HJW. Effects of iron limitation on photosynthesis and carbohydrate metabolism in the Antarctic diatom *Chaetoceros brevis* (Bacillariophyceae). *Eur. J. Phycol.* (2004) 39:161-171.

2. Leonardos N, Geider RJ. Effects of nitrate:phosphate supply ratio and irradiance on the C:N:P stoichiometry of *Chaetoceros muelleri*. Eur. *J. Phycol.* (2004) 39:173-180.

3. Hall SR, Leibold MA, Lytle DA, Val H. Smith Stoichiometry and Planktonic Grazer Composition over Gradients of Light, Nutrients, and Predation Risk. *Ecology* (2004) 85:2291-2301.

4. Hall SR, Smith VH, Lytle DA, Leibold MA. Constraints on primary producer N:P stoichiometry along N:P supply ratio gradients. *Ecology* (2005) 86:1894-1904.

5. Elser JJ, Frost P, Kyle M, Urabe J, Andersen T. Effects of light and nutrients on plankton stoichiometry and biomass in a P-limited lake. *Hydrobiologia* (2002) 481:101-112.

6. Dickman EM, Newell JM, González MJ, Vanni MJ. Light, nutrients, and food-chain length constrain planktonic energy transfer efficiency across multiple trophic levels. *Proc. Natl. Acad. Sci. U.S.A.* (2008) 105:18408-18412.

7. Spilling K, Ylöstalo P, Simis S, Seppälä J. Interaction Effects of Light, Temperature and Nutrient Limitations (N, P and Si) on Growth, Stoichiometry and Photosynthetic Parameters of the Cold-Water Diatom *Chaetoceros wighamii*. *PloS ONE* (2015) 10: e0126308.

8. Frost PC, Elser JJ. Effects of light and nutrients on the net accumulation and elemental composition of epilithon in boreal lakes. *Fresh. Biol.* (2002) 47:173-183.

9. Rosemond AD. Interactions among irradiance, nutrients, and herbivores constrain a stream algal community. *Oecologia* (1993) 94:585-594.

10. Hessen DO, Leu E, Færøvig PJ, Petersen SF. Light and spectral properties as determinants of C:N:P-ratios in phytoplankton. *Deep-Sea Res.* II (2008) 55:2169-2175.

11. Mette EM, Vanni MJ, Newell JM, González MJ. Phytoplankton communities and stoichiometry are interactively affected by light, nutrients, and fish. *Limnol. Oceanogr.* (2011) 56:1959-1975.

12. Liess A, Lange K, Schulz F, Piggott JJ, Matthaei CD, Townsend CR. Light, nutrients and grazing interact to determine diatom species richness via changes to productivity, nutrient state and grazer activity. *J. Ecol.* (2009) 97:326-336.

13. Dickman EM, Vanni MJ, Horgan MJ. Interactive effects of light and nutrients on phytoplankton stoichiometry. *Oecologia* (2006) 149:676-689.

14. Urabe J, Kyle M, Makino W, Yoshida T, Andersen T, Elser JJ. Reduced light increases herbivore production due to stoichiometric effects of light/nutrient balance. *Ecology* (2002) 83:619-627.

15. Urabe J, Sterner RW. Regulation of herbivore growth by the balance of light and nutrients. *Proc. Natl. Acad. Sci. U.S.A.* (1996) 93:8465-8469.

16. Hessen DO, Faerøvig PJ, Andersen T. Light, nutrients, and P:C ratios in algae: Grazer performance related to food quality and quantity. *Ecology* (2002) 83: 1886–1898.

17. Feng Y, Hare CE, Rose JM, Handy SM, DiTullio GR, Lee PA, et al. Interactive effects of iron, irradiance and CO_2_ on Ross Sea phytoplankton. *Deep-Sea Res. I* (2010) 57:368-383.

18. Moore CM, Hickmanb AE, Poultonb AJ, Seeyaveb S, Lucas MI. Iron–light interactions during the CROZet natural iron bloom and EXport experiment (CROZEX): II—Taxonomic responses and elemental stoichiometry. *Deep Sea Res. II* (2007) 54:2066-2084.

19. Thompson PA, Levasseur ME, Harrison PJ. Light-limited growth on ammonium vs. Nitrate: What is the advantage for marine phytoplankton? *Limnol. Oceanogr.* (1989) 34:1014-1024.

20. Plum C, Hüsener M, Hillebrand H. Multiple vs. single phytoplankton species alter stoichiometry of trophic interaction with zooplankton. *Ecology* (2015) 96:3075-3089.

21. Guo F, Kainz MJ, Valdez D, Sheldon F, Bunn SE. The effect of light and nutrients on algal food quality and their consequent effect on grazer growth in subtropical streams. *Freshw. Sci.* (2016) 35:1202-1212.

22. Guariento RD, Carneiro LS, Caliman A, Leal JJF, Bozelli RL, Esteves FA. Food web architecture and basal resources interact to determine biomass and stoichiometric cascades along a benthic food web. *PloS ONE* (2011) 6:e22205.

23. Sanches LF, Guariento RD, Caliman A, Bozelli RL, Esteves FA. Effects of nutrients and light on periphytic biomass and nutrient stoichiometry in a tropical black-water aquatic ecosystem. *Hydrobiologia* (2011) 669:35-44.

24. Liess A, Kahlert M. Gastropod grazers and nutrients, but not light, interact in determining periphytic algal diversity. *Oecologia* (2007) 152:101-111.

25. Rock AM, Hall MR, Vanni MJ, Gonzalez MJ. Carnivore identity mediates the effects of light and nutrients on aquatic food-chain efficiency. *Freshw. Biol.* (2016) 61:1492-1508.

26. Winston B, Scott JT, Pollock E. The synergistic effect of elevated CO_2_ and phosphorus on reservoir eutrophication. *Lake Reser. Manag.* (2016) 32:373-385. doi: 10.1080/10402381.2016.1231247

27. Li W, Gao K, Beardall J. Interactive effects of ocean acidification and nitrogen-limitation on the diatom *Phaeodactylum tricornutum*. *PLoS ONE* (2012) 7: e51590.

28. King AL, Sañudo-Wilhelmy SA, Leblanc K, Hutchins DA, Fu F. CO_2_ and vitamin B12 interactions determine bioactive trace metal requirements of a subarctic Pacific diatom. *ISME J.* (2011) 5:1388-1396.

29. Feng Y, Hare CE, Rose JM, Handy SM, DiTullio GR, Lee PA, et al. Interactive effects of iron, irradiance and CO_2_ on Ross Sea phytoplankton. *Deep-Sea Res.I* (2010) 57:368-383.

30. Leonardos N, Geider RJ. Elevated atmospheric carbon dioxide increases organic carbon fixation by *Emiliania huxleyi* (Haptophyta), under nutrient-limited high-light conditions. *J. Phycol.* (2005) 41:1196-1203.

31. Heiden JP, Bischof K, Trimborn S. Light intensity modulates the response of two antarctic diatom species to ocean acidification. *Front. Mar. Sci.* (2016) 3:260.

32. Shi D, Li W, Hopkinson BM, Hong H, Li D, Kao S-J, et al. Interactive effects of light, nitrogen source, and carbon dioxide on energy metabolism in the diatom *Thalassiosira pseudonana*. *Limnol. Oceanogr.* (2015) 60:1805-1822.

33. Feng Y, Warner ME, Zhang Y, Sun J, Fu F-X, Rose JM, et al. Interactive effects of increased pCO_2_, temperature and irradiance on the marine coccolithophore *Emiliania huxleyi* (Prymnesiophyceae). *Europ. J. Phycol.* (2008) 43:87-98.

34. Burkhardt S, Zondervan I, Riebesell U. Effect of CO_2_ concentration on C:N:P ratio in marine phytoplankton: A species comparison. *Limnol. Oceanogr.* (1999) 44: 683-690.

35. Frost PC, Cherrier CT, Larson JH, Bridgham S, Lamberti GA. Effects of dissolved organic matter and ultraviolet radiation on the accrual, stoichiometry and algal taxonomy of stream periphyton. *Fresh. Biol.* (2007) 52:319-330.

36. Xenopoulos MA, Frost PC, Elser JJ. Joint effects of UV radiation and phosphorus supply on algal growth rate and elemental composition. *Ecology* (2002) 83: 423-435.

37. Carrillo P, Delgado-Molina JA, Medina-Sánchez JM, Bullejos FJ, Villar-Argaiz M. Phosphorus inputs unmask negative effects of ultraviolet radiation on algae in a high mountain lake. *Glob. Change Biol.* (2008) 14:423-439.

38. Delgado-Molina JA, Carrillo P, Medina-Sánchez JM, Villar-Argaiz M, Bullejos FJ. Interactive effects of phosphorus loads and ambient ultraviolet radiation on the algal community in a high-mountain lake. *J. Plankton Res.* (2009) 31:619-634.

39. Hessen DO, Leu E, Færøvig PJ, Petersen SF. Light and spectral properties as determinants of C:N:P-ratios in phytoplankton. *Deep-Sea Res.* II (2008) 55:2169-2175.

40. Nielsen MV. Growth and chemical composition of the toxic dinoflagellate *Gymnodinium galatheanum* in relation to irradiance, temperature and salinity. *Mar. Ecol. Progr. Ser.* (1996) 136:205-211.

41. Wohlers-Zöllner J, Breithaupt P, Walther K, Jürgens K, Riebesell U. Temperature and nutrient stoichiometry interactively modulate organic matter cycling in a pelagic algal–bacterial community. *Limnol. Oceanogr.* (2011) 56:599-610.

42. Makino W, Gong Q, Urabe J. Stoichiometric effects of warming on herbivore growth: experimental test with plankters. *Ecosphere* (2011) 2:art79.

43. De Senerpont Domis LN, van De Waal DB, Helmsing NR, Van Donk E, Mooij WM. Community stoichiometry in a changing world: combined effects of warming and eutrophication on phytoplankton dynamics. *Ecology* (2014) 95:1485-1495.

44. Rhee G-Y, Gotham IJ.The effect of environmental factors on phytoplankton growth: Light and the interactions of light with nitrate limitation. *Limnol. Oceanogr.* (1981) 26: 649-659.

45. Moorthi SD, Schmitt JA, Ryabov A, Tsakalakis I, Blasius B, Prelle L, et al. Unifying ecological stoichiometry and metabolic theory to predict production and trophic transfer in a marine planktonic food web. *Phil. Trans. R. Soc. B* (2016) 371:20150270.

46. Weidman PR, Schindler DW, Thompson PL, Vinebrooke RD. Interactive effects of higher temperature and dissolved organic carbon on planktonic communities in fishless mountain lakes. *Freshw. Biol.* (2014) 59:889-904.

47. Hutchins DA, Fu F-X, Zhang Y, et al. 2007. CO_2_ control of Trichodesmium N_2_ fixation, photosynthesis, growth rates, and elemental ratios: implications for past, present, and future ocean biogeochemistry. *Limnol Oceanogr* 52: 1293–1304.

48. Fu F-X, Warner ME, Zhang Y, Feng Y, Hutchins DA. Effects of increased temperature and CO_2_ on photosynthesis, growth, and elemental ratios in marine *Synechococcus* and *Prochlorococcus* (cyanobacteria). *J. Phycol.* (2007) 43: 485-496.

49. Fu F-X, Mulholland MR, Garcia NS, Beck A, Bernhardt PW, Warner ME, et al. Interactions between changing pCO_2_, N_2_ fixation, and Fe limitation in the marine unicellular cyanobacterium *Crocosphaera*. *Limnol. Oceanogr.* (2008) 53:2472-2484.

50. Levitan O, Brown CM, Sudhaus S, Campbell D, LaRoche J, Berman-Frank I. Regulation of nitrogen metabolism in the marine diazotroph *Trichodesmium* IMS101 under varying temperatures and atmospheric CO_2_ concentrations. *Environm. Microbiol.* (2010) 12:1899-1912.

52. Stelzer RS, Lamberti GA. Effects of N:P ratio and total nutrient concentration on stream periphyton community structure, biomass, and elemental composition. *Limnol. Oceanogr.* (2001) 46:356-367.

53. Carrillo P, Medina-Sánchez JM, Herrera G, Durán C, Segovia M, Cortés D, et al. JM. 2015. Interactive effect of UVR and phosphorus on the coastal phytoplankton community of the western Mediterranean Sea: Unravelling eco-physiological mechanisms. *PloS ONE* (2015) 10:e0142987.

54. Passow U, Laws EA. Ocean acidification as one of multiple stressors: growth response of *Thalassiosira weissflogii* (diatom) under temperature and light stress. *Mar. Ecol. Progr. Ser.* (2015) 541:75-90.

55. Hoogstraten A, Timmermans KR, de Baar HJW. Morphological and physiological effects in *Proboscia alata* (Bacillariophyceae) grown under different light and CO_2_ conditions of the modern southern ocean. *J. Phycol.* (2012) 48:559-568.

56. Li G, Campbell DA. Rising CO_2_ Interacts with growth light and growth rate to alter photosystem II photoinactivation of the coastal diatom *Thalassiosira pseudonana*. *PloS One* (2013) 8:e55562.

57. Kranz SA, Levitan O, Richter K-U, Prásil O, Berman-Frank I, Rost B. Combined Effects of CO_2_ and Light on the N_2_-Fixing Cyanobacterium *Trichodesmium* IMS101: Physiological Responses. *Plant Physiol.* (2010) 154:334-345.

58. Taucher J, Jones J, James A, Brzezinski MA, Carlson CA, Riebesell U, et al. Combined effects of CO_2_ and temperature on carbon uptake and partitioning by the marine diatoms *Thalassiosira weissflogii* and *Dactyliosolen fragilissimus*. *Limnol. Oceanogr.* (2015) 60:901-919.

59. Sun J, Hutchins DA, Feng Y, Seubert EL, Caron DA, Fu F-X. Effects of changing pCO_2_ and phosphate availability on domoic acid production and physiology of the marine harmful bloom diatom *Pseudo-nitzschia* multiseries. *Limnol. Oceanogr.* (2011) 56:829-840.

60. Lefebvre SC, Benner I, Stillman JH, Parker AE, Drake MK, Rossignol PE, et al. Nitrogen source and pCO_2_ synergistically affect carbon allocation, growth and morphology of the coccolithophore *Emiliania huxleyi*: potential implications of ocean acidification for the carbon cycle. *Glob. Change Biol.* (2012) 18:493-503.

61. Korbee N, Carrillo P, Mata MT, Rosillo S, Medina-Sánchez JM, Figueroa FL. Effects of ultraviolet radiation and nutrients on the structure–function of phytoplankton in a hih mountain lake. *Photochem. Photobiol. Sci.* (2012) 11:1087-1098.

62. Villar-Argaiz M, Balseiro EG, Modenutti BE, Souza MS, Bullejos F, Medina-Sánchez JM, et al. Resource versus consumer regulation of phytoplankton: Testing the role of UVR in a Southern and Northern hemisphere lake. *Hydrobiologia* (2017) in press.

63. Bullejos FJ, Carrillo P, Gorokhova E, Medina-Sánchez JM, Balseiro EG, Villar-Argaiz M. Shifts in food quality for herbivorous consumer growth: multiple golden means in the life history. *Ecology* (2014) 95:1272-1284.

64. Ventura M, Liboriussen L, Lauridsen T, Søndergaard M, Søndergaard M, Jeppesen E. 2008. Effects of increased temperature and nutrient enrichment on the stoichiometry of primary producers and consumers in temperate shallow lakes. *Freshw. Biol.* 53, 1434-1452.

65. Paul C, Sommer U, Garzke J, Moustaka-Gouni M, Paul A, Matthiessen B. Effects of increased CO_2_ concentration on nutrient limited coastal summer plankton depend on temperature. *Limnol. Oceanogr.* (2016) 61:853-875.

66. Verspagen JM, van de Waal D B, Finke J F, Visser PM, Huisman J. Contrasting effects of rising CO_2_ on primary production and ecological stoichiometry at different nutrient levels *Ecol. Lett.* (2014) 17:951-960.
