# Supplementary material for: Predominant Non-additive Effects of Multiple Stressors on Autotroph C:N:P Ratios Propagate in Freshwater and Marine Food Webs
Source: Front Microbiol. 2018 Jan 30;9:69. doi: 10.3389/fmicb.2018.00069 (PMC5797581; doi:10.3389/fmicb.2018.00069)
Supplement: Supplementary file 9 [file Table3.DOCX]

**Figure legends for S1-S6**

**Figures S1-S6.** C:N:P-IEI for the 11 stressor pairs in this study. Numbers in the x-axis correponds to the number of the citation under supplementary references. Only one number is shown for studies with more than one observation that continue to the right. Color of bars indicate significant (black), not significant (white), not tested (grey) interactions in the original study. Gaps in the plots indicate absence of available data in the literature.

**Fig. S1=**  ↑ L x Nut, ↓ L x Nut

**Fig. S2**= ↑ CO_2_ x Nut, ↓ CO_2_ x Nut

**Fig. S3**= ↑ CO_2_ x Nut, ↓ CO_2_ x Nut

**Fig. S4**= ↑ T x Nut, ↓ T x Nut

**Fig. S5**= ↑ T x Sal, ↑ T x CO_2_, ↑ T x L, ↑ T x DOC

**Fig. S6**= UVA x Nut, UVB x Nut, UVR x Nut
